# Comparison of blood tonic efficacy and chemical constituents of *Kadsura interior* A.C. Smith and its closely related species

**DOI:** 10.1186/s13020-021-00544-w

**Published:** 2022-01-17

**Authors:** Jing Xu, Jiushi Liu, Bin Li, Xueping Wei, Yaodong Qi, Bengang Zhang, Haitao Liu, Peigen Xiao

**Affiliations:** 1 Key Laboratory of Bioactive Substances and Resources Utilization of Chinese Herbal Medicine, Ministry of Education, Institute of Medicinal Plant Development, Chinese Academy of Medical Sciences, Peking Union Medical College, Beijing, 100193 China; 2 Engineering Research Center of Traditional Chinese Medicine Resource, Ministry of Education, Institute of Medicinal Plant Development, Chinese Academy of Medical Sciences, Peking Union Medical College, Beijing, China

**Keywords:** Kadsurae Caulis, *Kadsura interior*, Blood tonic efficacy, Plant metabolomics, Spectrum-effect relationship

## Abstract

**Background:**

The stems of *Kadsura interior* A. C. Smith are used as traditional Chinese medicine (TCM) Kadsurae Caulis, with the traditional efficacy of tonifying and invigorating the blood, therefore being favored to treat blood deficiency (BD) widely. However, the stems of *K. interior* and its closely related species are morphologically similar and they may readily be misused as Kadsurae Caulis, thus likely to exert negative effects on clinical efficacy and clinical medication safety.

**Methods:**

Firstly, blood tonic efficacies of the stems of *K. interior* (KIS) and its closely related species were compared using BD mouse model induced by 1-acetyl-2-phenylhydrazine (APH) and cyclophosphamide (CTX). Secondly, the chemical constituents from the stems of *K. interior* and its closely related species were evaluated and compared using a plant metabolomics approach. Plant metabolomics in this study aims at discovering differential metabolites and comprehensively assessing the chemical constituents by combining state-of-the-art high-resolution UPLC-Q/TOF–MS/MS technique and multivariate data analysis. Finally, based on the pharmacological data and the chemical constituents in UPLC-Q/TOF–MS fingerprints, the potential blood tonic active markers were screened by the spectrum-effect relationship analysis and quantified by UPLC-UV-DAD.

**Results:**

The ethanol extract of the stems of *K. interior* significantly increased the levels of hematocrit (HCT), hemoglobin (HGB), and red blood cells (RBC) in BD mice. In addition, it significantly increased the serum levels of interleukin 3 (IL-3), granulocyte–macrophage colony-stimulating factor (GM-CSF), and macrophage-stimulating factor (M-CSF) in BD mice (*P* < 0.01). The blood tonic efficacy of the stems of *K. interior* was superior to those of its closely related species, especially at the dose of 200 mg/kg. Six differential compounds in the stems of *K. interior* were screened out to distinguish it from its closely related species. In combination with the results of the spectrum-effect relationship analysis, heteroclitin D, interiorin C, and heteroclitin G were identified as potential bioactive markers. The contents of heteroclitin D and heteroclitin G in the freeze-dried powder of KIS were 15.90 and 3.74 μg/mg.

**Conclusions:**

This study illustrated the differences in the blood tonic efficacies and the chemical constituents of the stems of *K. interior* and its closely related species, and pinpointed the potential bioactive markers of *K. interior*.

**Supplementary Information:**

The online version contains supplementary material available at 10.1186/s13020-021-00544-w.

## Background

The stems of *Kadsura* plants are popularly known as Chinese traditional folk medicines [1, 2]. Among them, *K. interior* A. C. Smith, the original plant of Kadsurae Caulis (*Dian Ji Xue Teng* in Chinese), was recorded in *Supplement to Compendium of Materia Medica* (*Ben Cao Gang Mu Shi Yi* in Chinese, published in 1765 A.D.) for the first time. Now it is officially documented in the current Chinese Pharmacopoeia (2020 edition, volume I). Due to its exceptional medicinal properties, Kadsurae Caulis is traditionally applied to tonify and invigorate blood in the TCM system, particularly to treat blood deficiency (BD) syndrome [3, 4]. Prior work on phylogenetic systematics revealed other three *Kadsura* species (*K. heteroclita* (Roxb.) Craib, *K. longipedunculata* Finet et Gagnep., and *K. japonica* (L.) Dunal) are closely related to *K. interior* [5]. Besides their genetic similarity, during the field research and market survey, we also discovered that they share indistinguishable morphological traits, such as leaves and stems. It could lead to their misuse in folk, thus potentially compromising the clinical efficacy of Kadsurae Caulis and even bringing underlying medication side effects [6, 7]. Hence, this study intended to investigate the distinction in the blood tonic efficacies and chemical constituents of the stems of *K. interior* and its closely related species, and also to identify the potential active markers of blood tonic activity.

BD syndrome is often accompanied by clinical symptoms of pallor, atrophy, weight loss, reduced function of important immune hematopoietic organs, comprising the spleen and the thymus [8–10]. Modern medicine has shown that BD syndrome embraced a wide range of anemia, including aplastic anemia, hemorrhagic anemia, and hemolytic anemia. The reduces of red blood cells (RBC), hemoglobin (HGB), hematocrit (HCT) concentration, and immunological function are common symptoms of these disorders [11]. The BD mouse model generated by 1-acetyl-2-phenylhydrazine (APH) coupled with cyclophosphamide (CTX) is commonly adopted to mimic the symptom of BD, such as weight loss, and decrease of blood routine indicators [12–15]. Therefore, blood routine indicators like HCT, HGB, and RBC were widely utilized to assess the blood tonic efficacy of drugs [16–18]. Besides, plant metabolomics enables the comprehensive comparison of the chemical constituents by combining the state-of-the-art high-resolution MS-based techniques with multivariate data analysis [19, 20]. Chemical analysis allows for the rapid identification of chemical constituents of TCM using UPLC-Q/TOF–MS/MS with and without standard substances [21, 22]. In recent years, the spectrum-effect relationship analysis has been commonly implemented to explore the bioactive markers of TCM, i.e., to evaluate the correlation between chemical constituents and pharmacological effects of TCM by data processing methods such as bivariate correlation analysis (BCA) and orthogonal partial least-squares regression analysis (OPLSR) [19, 23–25].

To explain whether the misuse of closely related species of *K. interior* as Kadsurae caulis is justified, a BD mouse model was combined with plant metabolomics to compare the blood tonic efficacies and chemical constituents of the stems of *K. interior* and its closely related species. Furthermore, the spectrum-effect relationship analysis based on BCA and OPLSR was employed to uncover the potential bioactive markers of the species that exerted advantageous blood tonic efficacy.

## Materials and methods

### Plant materials

The stems of *K. interior*, *K. heteroclita*, *K. longipedunculata*, *K. japonica* (KIS, KHS, KLS, KJS) were collected from different geographical origins in China (Table 1). The authentication of the voucher specimens was identified by Xinlei Zhao and Xueping Wei, researchers of the Institute of Medicinal Plant Development (IMPLAD), Beijing, China. The specimens were deposited in Medical Plant Resource Research Center in IMPLAD.

**Table 1:** Sample information for plant materials of four *Kadsura* species

No.	Species	Geographical origin
KI1	*K. interior*	Honghe hani and yi autonomous prefecture, Yunnan
KI2	*K. interior*	Honghe hani and yi autonomous prefecture, Yunnan
KI3*	*K. interior*	Lincang, Yunnan
KI4	*K. interior*	Lincang, Yunnan
KI5	*K. interior*	Lincang, Yunnan
KI6	*K. interior*	Lincang, Yunnan
KI7	*K. interior*	Lincang, Yunnan
KI8	*K. interior*	Lincang, Yunnan
KI9	*K. interior*	Lincang, Yunnan
KI10	*K. interior*	Lincang, Yunnan
KI11	*K. interior*	Lincang, Yunnan
KI12	*K. interior*	Lincang, Yunnan
KH1	*K. heteroclita*	Nanchuan, Chongqing
KH2	*K. heteroclita*	Qiandongnan Autonomous Prefecture, Guizhou
KH3	*K. heteroclita*	Laibin, Guangxi
KH4	*K. heteroclita*	Shaoguan, Guangdong
KH5	*K. heteroclita*	Shaoguan, Guangdong
KH6	*K. heteroclita*	Shaoguan, Guangdong
KH7	*K. heteroclita*	Shaoguan, Guangdong
KH8	*K. heteroclita*	Shaoguan, Guangdong
KH9	*K. heteroclita*	Shaoguan, Guangdong
KH10	*K. heteroclita*	Shaoguan, Guangdong
KH11	*K. heteroclita*	Shaoguan, Guangdong
KH12	*K. heteroclita*	Lushan, Jiangxi
KH13	*K. heteroclita*	Longyan, Fujian
KH14*	*K. heteroclita*	Dai Autonomous Prefecture of Xishuangbanna, Yunnan
KH15	*K. heteroclita*	Dai Autonomous Prefecture of Xishuangbanna, Yunnan
KH16	*K. heteroclita*	Dai Autonomous Prefecture of Xishuangbanna, Yunnan
KH17	*K. heteroclita*	Dai Autonomous Prefecture of Xishuangbanna, Yunnan
KH18	*K. heteroclita*	Dai Autonomous Prefecture of Xishuangbanna, Yunnan
KH19	*K. heteroclita*	Dai Autonomous Prefecture of Xishuangbanna, Yunnan
KH20	*K. heteroclita*	Dai Autonomous Prefecture of Xishuangbanna, Yunnan
KH21	*K. heteroclita*	Dai Autonomous Prefecture of Xishuangbanna, Yunnan
KH22	*K. heteroclita*	Dai Autonomous Prefecture of Xishuangbanna, Yunnan
KH23	*K. heteroclita*	Dai Autonomous Prefecture of Xishuangbanna, Yunnan
KH24	*K. heteroclita*	Dai Autonomous Prefecture of Xishuangbanna, Yunnan
KH25	*K. heteroclita*	Dai Autonomous Prefecture of Xishuangbanna, Yunnan
KH26	*K. heteroclita*	Hechi, Guangxi
KH27	*K. heteroclita*	Hechi, Guangxi
KL1	*K. longipedunculata*	Fuzhou, Fujian
KL2*	*K. longipedunculata*	Hangzhou, Zhejiang
KL3	*K. longipedunculata*	Huangshan, Anhui
KL4	*K. longipedunculata*	Huangshan, Anhui
KL5	*K. longipedunculata*	Huangshan, Anhui
KL6	*K. longipedunculata*	Huangshan, Anhui
KL7	*K. longipedunculata*	Longyan, Fujian
KL8	*K. longipedunculata*	Zhangping, Fujian
KL9	*K. longipedunculata*	Zhangping, Fujian
KL10	*K. longipedunculata*	Zhangping, Fujian
KJ1	*K. japonica*	Fuzhou, Fujian
KJ2	*K. japonica*	Fuzhou, Fujian
KJ3	*K. japonica*	Jianou, Fujian
KJ4	*K. japonica*	Longyan, Fujian
KJ5	*K. japonica*	Longyan, Fujian
KJ6	*K. japonica*	Nanping, Fujian
KJ7	*K. japonica*	Nanping, Fujian
KJ8*	*K. japonica*	Ningde, Fujian
KJ9	*K. japonica*	Sanming, Fujian
KJ10	*K. japonica*	Sanming, Fujian
KJ11	*K. japonica*	Sanming, Fujian
KJ12	*K. japonica*	Sanming, Fujian
KJ13	*K. japonica*	Wenzhou, Zhejiang
KJ14	*K. japonica*	Wuyishan, Fujian

Samples marked with * were used for the pharmacological experiments

### Drugs and reagents

UPLC-grade acetonitrile was acquired from Merck (Darmstadt, Germany). Pure water (18.2 MΩ) for UPLC analysis was generated with a Milli-Q water purification system (Millipore, Bedford, MA, USA). The other reagents of analytical grade were purchased from Beijing Chemical Corporation (Beijing, China). Standard substances 1–17 were isolated from the stems of *K. interior* in our previous studies (Unpublished observation), and standard substances 18–37 were bought from Tianjin Shilan Technology Co., Ltd. (Tianjin, China), Chengdu DeSiTe Biological Technology Co., Ltd. (Sichuan, China), Sichuan Weikeqi Biological Technology Co., Ltd. (Sichuan, China), and Chengdu Ruifensi Biotechnology Co., Ltd. (Sichuan, China) (Table 2). For all pharmacological experiments, the aqueous solutions of KIS, KHS, KLS and KJS were utilized at a concentration of 0.14 g/mL as the stock solutions. All dilutions were got from the stock solutions using a dilution vehicle that consisted of 0.3% CMC-Na. Fufang E’jiao Jiang (FEJ) was purchased from Dong’e E’jiao Co., Ltd. (Shandong, China), APH and CTX were purchased from Shanghai Yuanye Biotechnology Co., Ltd. (Shanghai, China), mouse interleukin 3 (IL-3), granulocyte–macrophage colony-stimulating factor (GM-CSF), macrophage-stimulating factor (M-CSF) Elisa kits were purchased from Jiangsu Enzyme Free Industry Co., Ltd. (Jiangsu, China).

**Table 2:** Information of the standard substances

No.	Compounds	Formula	Exact Mass	Information
1	Licarin A	C_20_H_22_O_4_	326.1518	Isolated from *K. interior*
2	Manwuwezic Acid	C_31_H_48_O_4_	484.3553	Isolated from *K. interior*
3	Interiorin C	C_24_H_26_O_8_	442.1628	Isolated from *K. interior*
4	Heteroclitin E	C_27_H_30_O_9_	498.1890	Isolated from *K. interior*
5	Kadsutherin F	C_28_H_28_O_8_	492.1784	Isolated from *K. interior*
6	Kadsutherin H	C_24_H_26_O_9_	458.1577	Isolated from *K. interior*
7	Kadsurin	C_25_H_30_O_8_	458.1941	Isolated from *K. interior*
8	Benzoyl Oxokadsuranol	C_29_H_28_O_9_	520.1733	Isolated from *K. interior*
9	( +)-Pinoresinol	C_20_H_22_O_6_	358.1416	Isolated from *K. interior*
10	Coumarinlignan	C_20_H_16_O_7_	368.0896	Isolated from *K. interior*
11	Vanillic Acid	C_8_H_8_O_4_	168.0423	Isolated from *K. interior*
12	Heteroclitin D	C_27_H_30_O_8_	482.1941	Isolated from *K. interior*
13	Prinsepiol	C_20_H_22_O_8_	390.1315	Isolated from *K. interior*
14	7-O-Methylcedrusin	C_20_H_24_O_6_	360.1573	Isolated from *K. interior*
15	Heteroclitin G	C_22_H_24_O_7_	400.1522	Isolated from *K. interior*
16	Schisantherin E	C_29_H_30_O_8_	506.1941	Isolated from *K. interior*
17	Manwuwezic Acid	C_31_H_48_O_4_	484.3553	Isolated from *K. interior*
18	Schisantherin A	C_30_H_32_O_9_	536.2046	58546-56-8^a^
19	Schisantherin B	C_28_H_34_O_9_	514.2203	DST190819-009^b^
20	Schisantherin E	C_30_H_34_O_9_	538.2203	DST190702-034^b^
21	Schisandrol A	C_24_H_32_O_7_	432.2148	7432-28-2^a^
22	Schisandrol B	C_23_H_28_O_7_	416.1835	58546-54-6^a^
23	Schizandrin A	C_24_H_32_O_6_	416.2199	DST190329-012^b^
24	Schizandrin B	C_23_H_28_O_6_	400.1886	DST190819-009^b^
25	Schizandrin C	C_22_H_24_O_6_	384.1573	DST190122-014^b^
26	Schisanhenol	C_23_H_30_O_6_	402.2042	DST190623-015^b^
27	Anwuligan	C_20_H_24_O_4_	328.1675	wkq16122106^c^
28	Chicanine	C_20_H_22_O_5_	342.1467	DST190904-051^b^
29	D-Epigalbacin	C_20_H_20_O_5_	340.1311	B-119-190723^d^
30	Gomisin D	C_28_H_34_O_10_	530.2152	wkq16032405^c^
31	Gomisin G	C_30_H_32_O_9_	536.2046	62956-48-3^a^
32	Gomisin H	C_23_H_30_O_7_	418.1992	DST190708-052^b^
33	Gomisin J	C_22_H_28_O_6_	388.1886	66280-25-9^a^
34	Gomisin O	C_23_H_28_O_7_	416.1835	DST190814-053^b^
35	Benzoylgomisin O	C_30_H_32_O_8_	520.2097	DST190806-087^b^
36	Angeloylgomisin O	C_28_H_34_O_8_	498.2254	DST190712-175^b^
37	Gomisin N	C_23_H_28_O_6_	400.1886	DST190806-037^b^

^a^Tianjin Shilan Technology Co., Ltd

^b^Chengdu Desite Biological Technology Co., Ltd

^c^Sichuan Weikeqi Biological Technology Co., Ltd

^d^Chengdu Ruifensi Biotechnology Co., Ltd

### Experimental animals

Male Kunming mice (18–22 g, SPF) were obtained from SPF Biotechnology Co., Ltd. (Beijing, China) (License number: SCXK [Beijing] 2019-0010). All mice were kept in a 12 h light/dark and temperature-controlled room, and were fed adaptively for 7 days with free access to food and water. All animal care and experimental processes were carried out following the National Institutes of Health guide.

### Pharmacological experiment on BD mice

#### Drug preparation

500 g powder (60 mesh) of KIS, KHS, KLS, KJS were weighed and soaked overnight in 10 volumes of 95% ethanol, then refluxed for 4 h for 5 times. The ethanol extracts were concentrated under pressure and freeze-dried with BUCHI Lyovapor™ L-200 (Büchi Labortechnik AG, Flawil, Switzerland) for use.

#### Model establishment and administration

The BD mouse model was induced by APH combined with CTX [9]. Mice were randomly divided into 11 groups (n = 10). All groups were: (1) Control group; (2) Model group; (3) Positive group (8 mL/kg FEJ); (4) KIS-L group (low dose, 200 mg/kg KIS); (5) KIS-H group (high dose, 400 mg/kg KIS); (6) KHS-L group (200 mg/kg KHS); (7) KHS-H group (400 mg/kg KHS); (8) KLS-L group (200 mg/kg KLS); (9) KLS-H group (400 mg/kg KLS); (10) KJS-L group (200 mg/kg KJS); (11) KJS-H group (400 mg/kg KJS). Model group and administration groups were subcutaneously injected (s.c.) with 2% APH saline on the 2nd day at a dose of 20 mg/kg, and 2% APH saline (s.c.) on the 5th day at a dose of 40 mg/kg, 4 h later, they were intraperitoneally injected (i.p.) with CTX saline at a dose of 40 mg/kg, and CTX saline was given once daily for the next three days (i.p.) at a dose of 40 mg/kg (6-8th days). Simultaneously, the control group was given an equal volume of normal saline (s.c. and i.p., correspondingly). From the first day of modelling, the FEJ group (8 mL/kg) and the low and high doses of KIS, KHS, KLS, and KJS groups (200, 400 mg/kg) were administered by gavage for 14 consecutive days with corresponding doses of drugs, once daily. The control group was administered with an equal volume of 0.3% CMC-Na by gavage at the same time.

#### Sample collection

The body weights of all mice were recorded daily before administration. An hour after the last administration, the mice were anesthetized with a small amount of diethyl ether. Two blood samples were taken by posterior orbital venous plexus approach for blood routine test and cytokine assays, with 1.5 mL EP tubes containing ethylene diamine tetraacetic acid (EDTA) and normal 1.5 mL EP tubes separately. The first blood sample was analyzed by a Sysmex XS-800i hematology analyzer (Sysmex Corporation, Japan) for peripheral hemogram analysis to measure HCT, HGB, and RBC. Supernatants from the other blood samples were taken and reserved until analysis. The thymus and spleen were fetched and weighed. Thymus index and spleen index were calculated as follows: Organ index = Organ weight (mg) / Body weight (g).

### Statistical analysis

Data were analyzed with GraphPad Prism (version 8.0.2, GraphPad Software, La Jolla, CA, USA). Statistical significance was assessed by one-way analysis of variances (ANOVA). The results were expressed as mean ± standard deviation (SD). *P* < 0.05 indicated significant differences.

### UPLC-Q/TOF–MS/MS analysis

#### Preparation of standard solution

Standard solutions (1 μg/mL) were prepared by dilution of stock solutions of each standard substance (0.1 mg/mL in methanol).

#### UPLC-Q/TOF–MS/MS conditions

Instrumental analysis was performed using UPLC on the Waters ACQUITY UPLC™ system (Waters Corporation, Milford, MA, USA), consisting of a binary solvent delivery manager, an autosampler, and a PDA detector. Chromatographic separations were performed on a Waters ACQUITY CORTECS C_18_ column (100 mm × 2.1 mm, 1.6 μm). The temperatures of column and auto-sampler were maintained at 25 °C and 10 °C, respectively. The flow rate was set at 0.3 mL/min. The binary gradient elution system including H_2_O (A) and acetonitrile (B) was applied with the flowing gradient program: 0–4 min, 33–36% B; 4–5 min, 36–45% B; 5–9 min, 45% B; 9–12 min, 45–50% B; 12–16 min, 50–56% B; 16–22 min, 56–70% B; 22–30 min, 70–95% B. The injection volume was 1 μL. The detection wavelength was set at 215 nm. The development of extraction and UPLC method for qualitative analysis was conducted (Details are listed in Additional file 1: Table S1, S2; Fig. S1). Mass spectrometric analysis was conducted on the Waters Xevo G2-XS Q/TOF mass spectrometer (Waters Corporation, Milford, MA, USA) in positive mode of the electrospray ionization (ESI) interface. The desolvation gas flow rate was set to 900 L/h at 300 °C. The cone gas was set to 50 L/h and the source temperature was set at 100 °C. The capillary and cone voltages were set at 3.5 kV and 30 V, respectively. MS/MS fragment information was obtained using a collision energy ramp from 20 to 40 V. Mass spectrometry was performed in full scan mode from *m/z* 50 to 1200. Leucine enkephalin (200 pg/mL infused at 20 mL/min) was used as a lock mass for mass correction ([M + H] ^+^ *m/z* 556.2771). Accurate mass and fragment ions were got using MassLynx™ software (version 4.1, Waters Corporation, Milford, MA, USA).

#### Construction of the In-House Database of *K. interior* and its related species

An in-house database of compounds from the genus *Kadsura* was constructed with the Progenesis SDF Studio. The compounds previously reported from *Kadsura* species were collected using search terms such as “*Kadsura*” in electronic sources such as Google Scholar, Web of Science, and CNKI. Additionally, the selected compounds (found in the articles) and their files (.mol) were determined by searching the compound name or structure in SciFinder. Last, with Progenesis SDF Studio, all files (.mol) describing the structures of the compounds were imported into an in-house database.

### Data processing

The acquired mass data were imported to Progenesis QI (Waters Corporation, Milford, MA, USA) for peak detection, alignment, deconvolution, peak picking, and normalization. Then a three-dimensional data matrix was output composed of the sample name, peak number (t_R_-*m*/*z* pair), and ion intensity. Finally, the resulting matrix was imported into SIMCA (version 14.1, Umetrics AB, Umeå, Sweden) for multivariate statistical analysis such as principal component analysis (PCA) and orthogonal partial least squares discriminant analysis (OPLS-DA) to classify the metabolic phenotypes. The data quality control was completed with SIMCA (Details are listed in Additional file 1). The loading plot from OPLS-DA together with the variable importance in the projection (VIP) was used to discover the potential differential compounds. Hierarchical cluster analysis (HCA) was conducted to estimate the consistency of these drugs. The HCA heatmap analysis shows the change in the content of all ions in each sample by a gradient of color change (blue-white-red).

### Spectrum-effect relationship analysis

Microsoft Excel ™ 2016 (Microsoft, USA) was used for BCA of the two groups of variables. SIMCA (version 14.1, Umetrics AB, Umeå, Sweden) was used for OPLSR. OPLSR was used to analyze the correlation between the chromatographic peak areas in UPLC-Q/TOF–MS fingerprints and the main pharmacological indicators. SIMCA (version 14.1, Umetrics AB, Umeå, Sweden) was used for OPLSR.

## Results

### Pharmacological effects

#### Body weights and general observations of mice

**Fig. 1:**
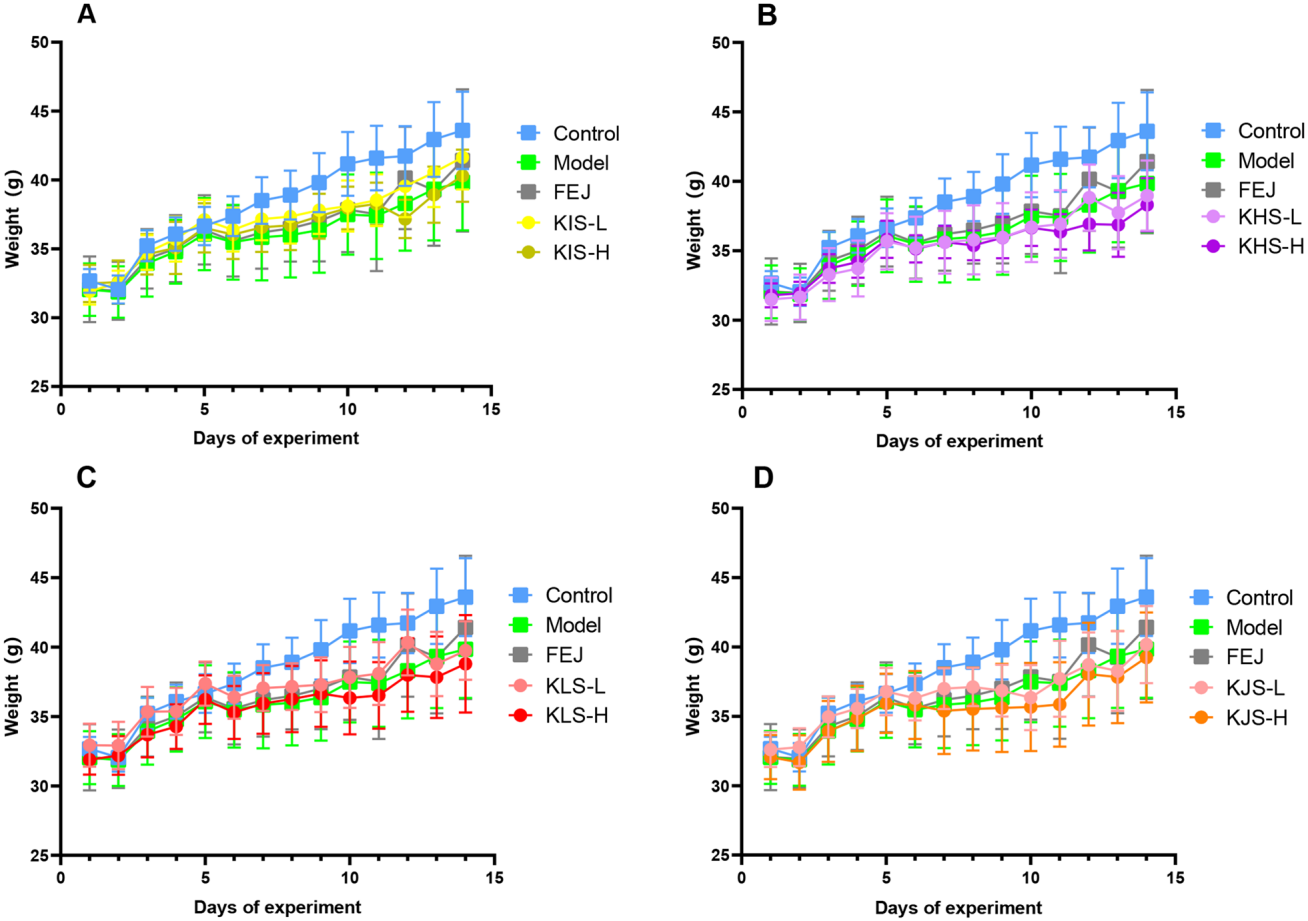
Body weight records of control, model, and all administration groups during the experiment for 14 days (mean ± SD, n = 10). **A** KIS groups, **B** KHS groups, **C** KLS groups, **D** KJS groups

The mice in the model group exhibited poor mental status, dry and sparse hair, exhausted with pale paw color, lip color, and tail color. It appeared that the BD mouse model was successfully established. The body weights of the mice in the control group steadily increased, whereas the body weights of the mice in other groups decreased in the first 5 days, and gradually increased in varying degrees after stopping modelling at the 8th day (Fig. 1), with the body weights of mice in the model group increasing at the slowest rate. Additionally, the body weight changes were also related to behavioral changes. The mice in the FEJ and KIS groups had considerably higher body weights and were more energetic, with dense and shiny fur, pink and moist noses and lips, rounded and pink tails, and a better appetite.

#### Routine blood analysis

**Fig. 2:**
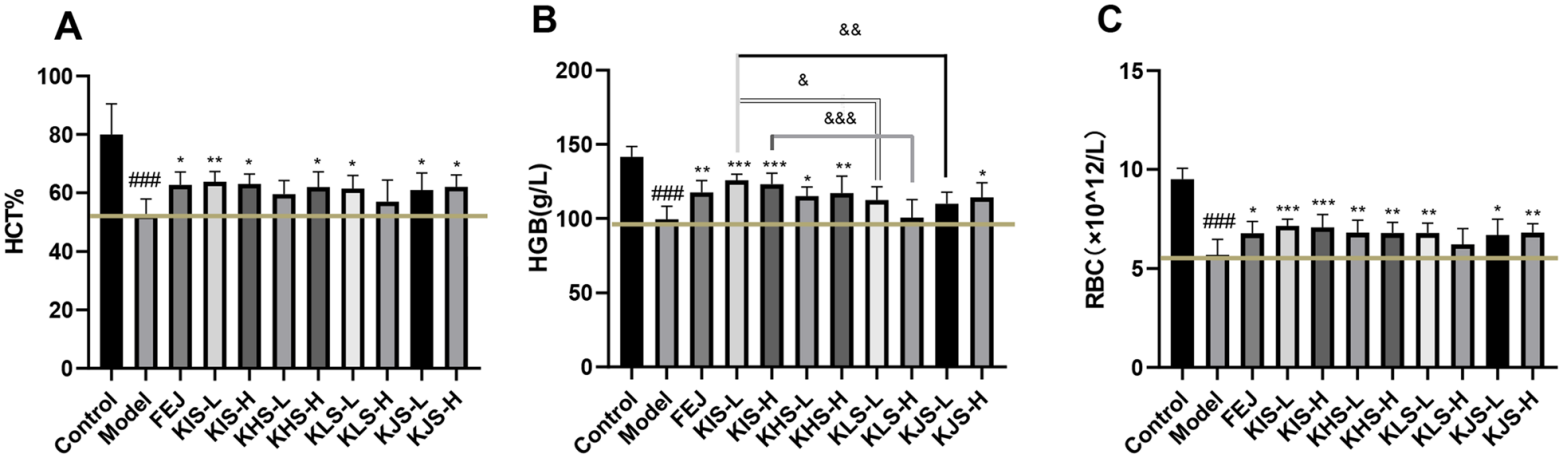
Effects of FEJ, KIS, KHS, KLS, and KJS on the blood routine indicators of BD mice (mean ± SD, n = 10). ^###^*P* < 0.001 vs control group, *P < 0.05, **P < 0.01, ***P < 0.001 vs model group. & *P* < 0.05, && *P* < 0.01, &&&* P* < 0.001, differences between connected groups. **A** Effect on HCT. **B** Effect on HGB. **C** Effect on RBC

Peripheral blood cell levels can directly reflect the status of the hematopoietic function. After the administration for 14 days, compared with the control group, the HCT, HGB, RBC levels in the model group mice decreased significantly (*P* < 0.001), indicating that the BD mouse model was successfully induced. After the administration with FEJ, KIS, KHS, KJS, all the above indicators showed an increasing trend (Fig. 2), compared with the model group, HGB and RBC levels in mice administered with two doses of KIS were increased significantly (*P* < 0.001), and the HCT levels of the low-dose KIS were increased significantly (*P* < 0.01). However, high-dose KHS significantly improved HCT, HGB, and RBC levels (*P* < 0.05, *P* < 0.01, *P* < 0.01), but there was no significant difference in HCT levels with low-dose KHS compared to the model group, and for KLS, there was no significant difference in improving HCT, HGB, and RBC in the high-dose group, and for KJS, high-dose group significantly improved HCT, HGB, and RBC (*P* < 0.05, *P* < 0.05, *P* < 0.01). In addition, the average of these indicators in groups treated with KIS were higher than those with other herbs, the error bars of KIS groups were lowest, showing that KIS had a more stable blood tonic efficacy [26, 27]. According to the results of the study, KIS could better improve the haematopoietic effect of chemotherapy-induced BD syndrome in mice.

#### Changes in organ index

**Fig. 3:**
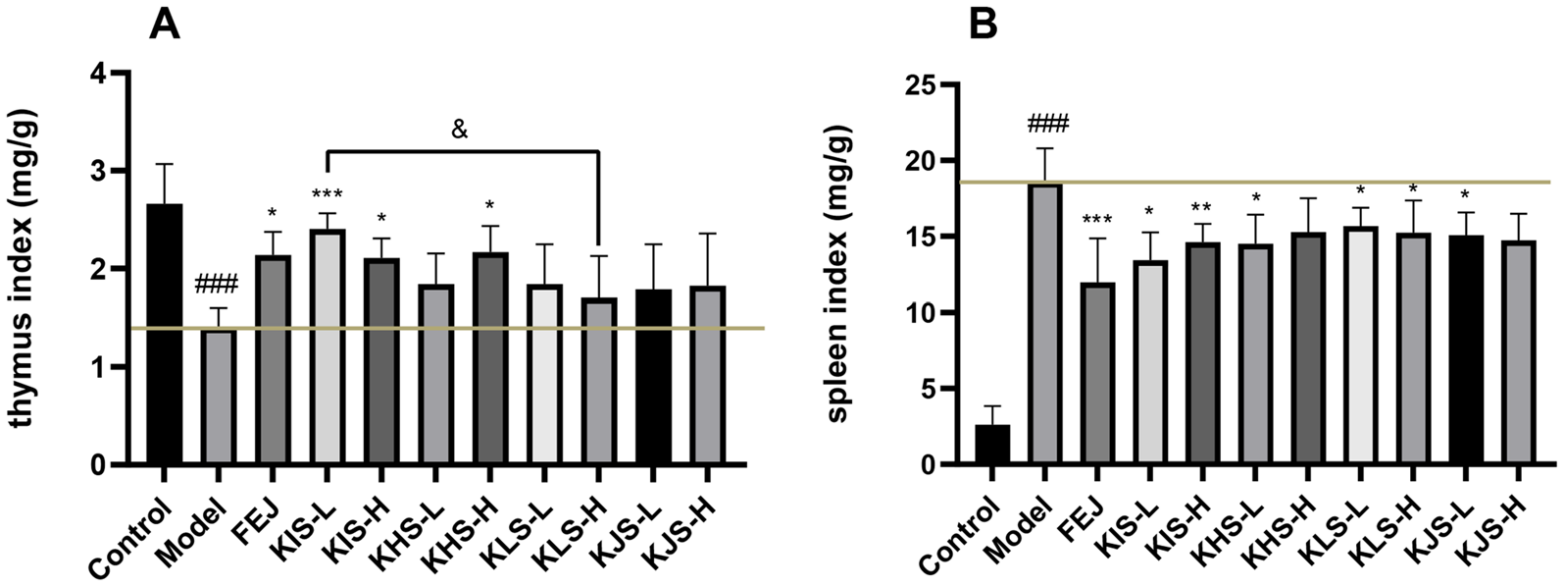
Effects of FEJ, KIS, KHS, KLS, and KJS on the thymus index and spleen index (mean ± SD, n = 10). ^###^*P* < 0.001 vs control group, *P < 0.05, **P < 0.01, ***P < 0.001 vs model group. &* P* < 0.05, differences between connected groups. **A** Effect on the thymus index in mice. **B** Effect on the spleen index in mice

As shown in Fig. 3, the thymus index of model group mice decreased significantly compared to the control group (*P* < 0.001) while the spleen index showed a compensatory increase significantly compared to the control group (*P* < 0.001). Compared to the model group, the thymus index of mice in FEJ group, KIS groups, and KHS-H group were all increased significantly (*P* < 0.05), while the KIS-L group was improved more obviously than KIS-H and KHS-H group. Among them, the thymus index of group treated with low-dose KIS was significantly higher than that with low-dose of KLS (*P* < 0.05), spleen index of mice in FEJ group and KIS-L group all decreased more significantly than other administration groups (*P* < 0.05), while the KLS-L group was not significantly improved. The extracts of the stems of *K. interior* and its closely related species were compared and their effects on thymus index and spleen index were as follows: KIS groups > KHS groups > KJS groups > KLS groups (*P* < 0.01).

#### Effect on the serum levels of IL-3, GM-CSF, and M-CSF

Based on the above results that KIS and KHS exerted better blood tonic properties, the effects of KIS and KHS on serum levels of important hemopoietic growth factors like IL-3, GM-SCF, and M-CSF were further investigated. The serum levels of IL-3, GM-CSF, and M-CSF of the control group, model group, FEJ group, KIS groups and KHS groups were measured using a spark multimode microplate reader (Tecan, Switzerland) according to the kit instructions.

**Fig. 4:**
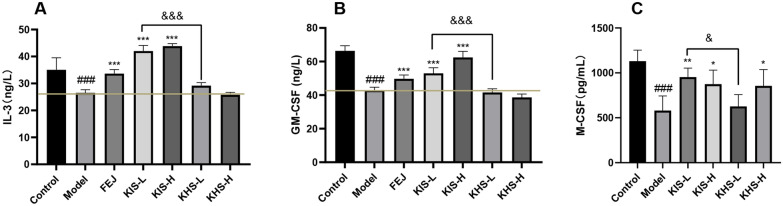
Effect of KIS, KHS on IL-3, GM-CSF, and M-CSF in serum of BD mice (mean ± SD, n = 10). ^###^*P* < 0.001 vs control group, ***P < 0.001 vs model group. &* P* < 0.05, && *P* < 0.01, &&& *P* < 0.001, differences between connected groups. **A** Effect on serum IL-3. **B** Effect on serum GM-CSF. **C** Effect on serum M-CSF

The serum levels of IL-3, GM-CSF, and M-CSF significantly decreased in the model group compared with the control group (*P* < 0.001). Compared with the model group, the serum levels of IL-3, GM-CSF, and M-CSF of mice treated with KIS significantly increased (*P* < 0.001; *P* < 0.01). No improvement in IL-3 and GM-CSF levels of mice administered with KHS extracts was observed. Whereas, the serum level of M-CSF of mice administered with KHS-H was increased. This suggested that the improvement of KIS on BD mice was achieved by elevating IL-3, GM-CSF, and M-CSF while KHS only effected on the serum level of M-CSF (Fig. 4).

### Chemical analysis

#### Compound identification of these four *Kadsura* crude drugs

**Fig. 5:**
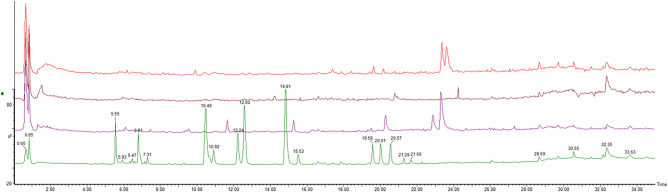
TIC of four *Kadsura* crude drugs. From bottom to top: *K. interior*, *K. heteroclita*, *K. longipedunculata*, *K. japonica*

Total ion current (TIC) chromatograms of four *Kadsura* crude drugs were obtained by UPLC-Q/TOF–MS/MS method (Fig. 5). From the TIC chromatograms, the chemical constituents of four *Kadsura* crude drugs show significant differences. To further investigate the chemical constituents of these four *Kadsura* crude drugs, the retention time, precise molecular weight, and secondary mass spectrometric cleavage fragment information of the peaks of each chemical component were compared with the standard substances and the in-house library: 20 compounds were identified, and 36 compounds were inferred by comparison with the in-house database (Table 3). They included 48 lignans, 5 triterpenoids, 1 sesquiterpenoid, 1 phenolic acid, and 1 phenolic compound.

**Table 3:** Compounds identified in four *Kadsura* species using UPLC-Q/TOF–MS/MS

No.	Compounds	Rt (min)	Fomula	Error (× 10^–6^)	Measured [M + H] +	Major fragments	Calculated *m/z*	Structural type	KIS	KHS	KLS	KJS
					(Measured [M + Na] +)[Measured [2 M + Na]+]							
1*	Vanillic Acid	0.96	C_8_H_8_O_4_	− 4.17	169.0494	151.0385, 141,9584	168.0423	phenolic acids	Y↓	Y↑	Y	Y
2*	7-O-Methylcedrusin	1.35	C_20_H_24_O_6_	− 2.50	361.1642	331.1591, 151.0762	360.1573	phenolics	Y	Y	Y	Y
3*	( +)-Pinoresinol	1.98	C_20_H_22_O_6_	4.18	359.1509	342.1393, 323.1288, 313.1444	358.1416	lignans	Y↓	Y	Y↑	Y
4*	Kadsutherin H	3.7	C_24_H_26_O_9_	0	459.1655	399.1460, 371.1475, 339.1221	458.1577	lignans	Y	/	/	/
5	Micrandilactone I	4.53	C_30_H_44_O_7_	0.17	517.3161		516.3087	triterpenoids	/	/	Y	/
6	Kadsuranin	4.87	C_23_H_28_O_6_	− 1.12	401.1954		400.1886	lignans	Y↓	Y	Y↑	Y
7*	Coumarinlignan	4.89	C_20_H_16_O_7_	− 1.90	369.0967	351.0896, 321.0761, 203.0324	368.0896	lignans	Y	Y	/	/
8	Schisantherin G	4.99	C_29_H_34_O_11_	2.34	559.2187		588.2101	lignans	/	/	Y	Y
9	Kadoblongifolin B	5.43	C_22_H_24_O_8_	1.92	417.1557	399.1437, 382.3145	416.1471	lignans	Y	Y	Y↑	Y↓
10*	Heteroclitin G	5.57	C_22_H_24_O_7_	− 0.75	401.1597	383.1509, 341.1031, 313.1082	400.1522	lignans	Y↑	Y	Y	Y↓
11*	Kadsutherin F	5.96	C_28_H_28_O_8_	4.47	493.1884	403.1661, 401.1638,383.1509	492.1784	lignans	Y	/	/	/
12*	Schisandrol A	6.22	C_24_H_32_O_7_	3.93	(455.2063)	415.2180, 384.1929	432.2148	lignans	Y	Y	Y	Y
13	Angeloylgomisin M1	6.28	C_27_H_32_O_8_	4.13	485.2195	401.1617, 385.1644, 371.1505	484.2097	lignans	Y↑	Y↓	Y	Y
14*	Interiorin C	6.35	C_24_H_26_O_8_	4.98	443.1728	383.1509, 355.1547	442.1628	lignans	Y↑	Y	Y	Y
15*	Heteroclitin E	6.49	C_27_H_30_O_9_	0.80	499.1972	399.1450, 371.1515, 339.1221	498.189	lignans	Y	Y	Y	/
16*	Benzoyl Oxokadsuranol	6.64	C_29_H_28_O_9_	− 1.92	521.1801	503.1685, 399.1460, 371.1475	520.1733	lignans	Y	Y	Y	Y
17*	Schisantherin E	6.96	C_30_H_34_O_9_	− 4.27	(561.2078)	417.1923, 373.1645	538.2203	lignans	/	/	Y	/
18	Longipedlignan H	6.97	C_24_H_26_O_9_	− 1.53	459.1648	399.1460, 355.1161, 327.1205	458.1577	lignans	Y	Y↑	Y↓	Y
19	Longipedlignan E	7.03	C_27_H_32_O_9_	2.26	[1023.4008]		500.2046	lignans	/	Y	/	/
20	4β,9β-dihydroxy-1α,5α-H-guaia-6,10 (14)-dien	7.07	C_15_H_24_O_2_	1.69	236.178	219.1757, 194.1159	236.1776	sesquiterpenoids	Y	Y↑	Y	Y↓
21	Acetoxyl Oxokadsurane	7.63	C_24_H_26_O_8_	2.26	443.1688	383.1468, 355.1547	442.16	lignans	/	/	Y	/
22	Longipedlactone C	7.66	C_30_H_40_O_6_	3.31	497.2914		496.2825	triterpenoids	/	Y	Y	Y
23	Piperitol	7.8	C_20_H_20_O_6_	0	357.1338	311.0954, 222.1118	356.126	lignans	Y	Y↓	Y↑	Y
24	Kadoblongifolin A, Kadoblongifolin B	8.65	C_22_H_24_O_8_	− 0.48	417.1547	385.1685, 354.2850, 315.0891	416.1471	lignans	Y	Y	Y↓	Y↑
25	Schizanrin M	8.75	C_22_H_24_O_7_	0.50	401.1602	383.1576, 354.2850, 186.0572	400.1522	lignans	Y	Y	Y↑	Y↓
26	6-hydroxyhinokinin	8.85	C_20_H_18_O_7_	3.24	(393.0963)	357.1370, 325.1088	370.1053	lignans	Y↓	Y↑	Y	Y
27	Interiotherin B	9.01	C_27_H_30_O_9_	0.80	499.1972	399.1437, 382.3145, 355.1175, 325.1088, 279.0919	498.189	lignans	Y↑	Y↓	Y	Y
28	Angeloylbinankadsurin A	9.21	C_27_H_32_O_8_	− 0.41	485.2186	385.1644, 354.2850	484.211	lignans	Y↓	Y	Y	Y↑
29	Gomisin J	9.67	C_22_H_28_O_6_	− 2.65	389.1948		388.1886	lignans	Y	Y	/	Y
30	Kadsulignan I	10.34	C_25_H_28_O_8_	1.32	457.1868	371.1492, 356.1256	456.1784	lignans	Y↑	Y↓	Y	Y
31	Angeloylgomisin R	10.49	C_27_H_30_O_8_	0	483.2019	383.1492, 368.1260	482.1941	lignans	Y↑	Y	Y↓	Y
32	Interiorin D	10.92	C_29_H_28_O_8_	− 0.99	505.1861	497.1571	504.1788	lignans	Y↑	Y	Y↓	Y
33	Heteroclitin O	11.22	C_34_H_32_O_11_	2.84	617.2035		616.1945	lignans	/	/	Y	/
34	Longipedlignan I	11.48	C_26_H_30_O_9_	2.41	486.1902n		486.189	lignans	Y	Y	Y	Y
35	Schizanrin F	11.51	C_32_H_34_O_11_	1.19	595.2181		594.2101	lignans	Y↓	Y	Y↑	Y
36	Kadnanolactone A	11.65	C_30_H_38_O_5_	1.25	479.2803	432.2034, 415.1756	478.2719	triterpenoids	Y↓	Y↑	Y	Y
37	( +)-Gomisin K3	12.04	C_23_H_30_O_6_	0.62	403.2118		402.2042	lignans	Y	Y	Y	Y
38	Heteroclitin C, Heteroclitin B	12.11	C_28_H_34_O_8_	3.38	(521.2163)		498.2254	lignans	Y	Y↓	Y↑	Y
39	Kadlongilignan E	12.34	C_27_H_30_O_9_	− 0.20	499.1967	483.2374, 399.1437, 369.1348	498.189	lignans	/	Y↓	Y↑	/
40*	Heteroclitin D	12.65	C_27_H_30_O_8_	− 4.77	483.1996	383.1549, 366.1547, 323.1251	482.1941	lignans	Y↑	Y	Y	Y↓
41	Longipedlactone B	13.2	C_30_H_40_O_5_	1.62	481.2956		480.2876	triterpenoids	/	Y	Y	Y↑
42	Licarin A	13.36	C_20_H_22_O_4_	− 4.91	327.158	270.3151, 229.1418, 182.9854	326.1518	lignans	Y	Y	Y	Y
43*	Schisantherin A	13.43	C_30_H_32_O_9_	− 2.05	(559.1933)	415.1763, 371.1475, 340.1289	536.2046	lignans	/	Y↑	Y↓	Y
44*	Schisantherin B	13.45	C_28_H_34_O_9_	1.36	(537.2108)	415.1805, 371.1475	514.2203	lignans	/	Y	Y	Y
45*	Kadsurin	15.53	C_25_H_30_O_8_	− 4.36	459.1999	400.1850, 369.1675, 354.1414	458.1941	lignans	Y	/	/	/
46	Isoanwulignan, ( +)-anwulignan	16.38	C_20_H_24_O_4_	3.00	(351.1577)		328.1675	lignans	Y	Y	Y	Y
47	Interiotherin C	16.75	C_30_H_36_O_10_	4.42	(579.2225)	457.1851, 357.1331	556.2308	lignans	Y	Y	Y	/
48	Gomisin A (Schisandrol B), Gomisin O	16.84	C_23_H_28_O_7_	3.87	417.1924		416.1835	lignans	Y↓	Y↑	Y	Y
49	Schiarisanrin D	16.98	C_31_H_30_O_8_	1.13	531.2025	383.1471, 355.1522, 186.0572	530.1941	lignans	Y	Y	Y	/
50*	D-Epigalbacin	18.16	C_20_H_20_O_5_	− 4.70	341.1373	323.1269, 302.3088, 279.0953	340.1311	lignans	/	/	Y	/
51	Schizandrin	18.2	C_24_H_32_O_7_	0	(455.2031)	385.1644, 354.2889, 279.0919	432.2148	lignans	/	Y	/	/
52*	Gomisin N	20.07	C_23_H_28_O_6_	0.50	401.1966	370.1750, 355.1541	400.1886	lignans	Y↑	Y	Y	Y
53	Schizandrin C	21.25	C_22_H_24_O_6_	3.64	(407.1485)	385.1641, 301.1434	384.1573	lignans	/	/	Y	/
54	Heteroclitin A, Caproylbinankadsurin A	22.02	C_28_H_36_O_8_	4.23	(523.2324)		500.241	lignans	/	Y	/	/
55*	Manwuwezic Acid	26.21	C_30_H_46_O_4_	4.46	471.3495	453.3355	470.3396	triterpenoids	Y	Y	Y	Y
56*	Prinsepiol	29.77	C_20_H_22_O_8_	2.05	391.1401	371.3170, 284.2935, 279.1594	390.1315	lignans	Y	Y	Y	Y

Compounds marked with * were identified with standard compound, other 36 compounds were identified with the in-house database

Y: exist; /: don’t exist; ↑: significantly highest content; ↓: with lowest content

Besides 56 compounds identified and inferred from the compound database of *Kadsura* genus, other 70 compounds were identified with a natural compound database. An extensive description of the identified compounds is shown in Table 3 in Additional file 1, including their adducts, molecular formula, confidence score, fragmentation score, mass error, and isotope similarity. These compounds were classified according to their structures: 17 alkaloids; 1 coumarin; 2 fatty acids; 19 flavonoids; 7 glycosides; 6 lignans; 2 lipids; 3 phenolic acid and derivatives; 1 saponin; 1 terpenoid; 3 others and 8 compounds with unknown structures still need to be determined. In addition, among these compounds, the structures of nine pairs of isomers need to be confirmed. These compounds were reported from *K. interior* and its related species for the first time. In conclusion of this study, lignans are main compounds identified from the stems of *K. interior* and its closely related species.

#### Multivariate statistical analysis

**Fig. 6:**
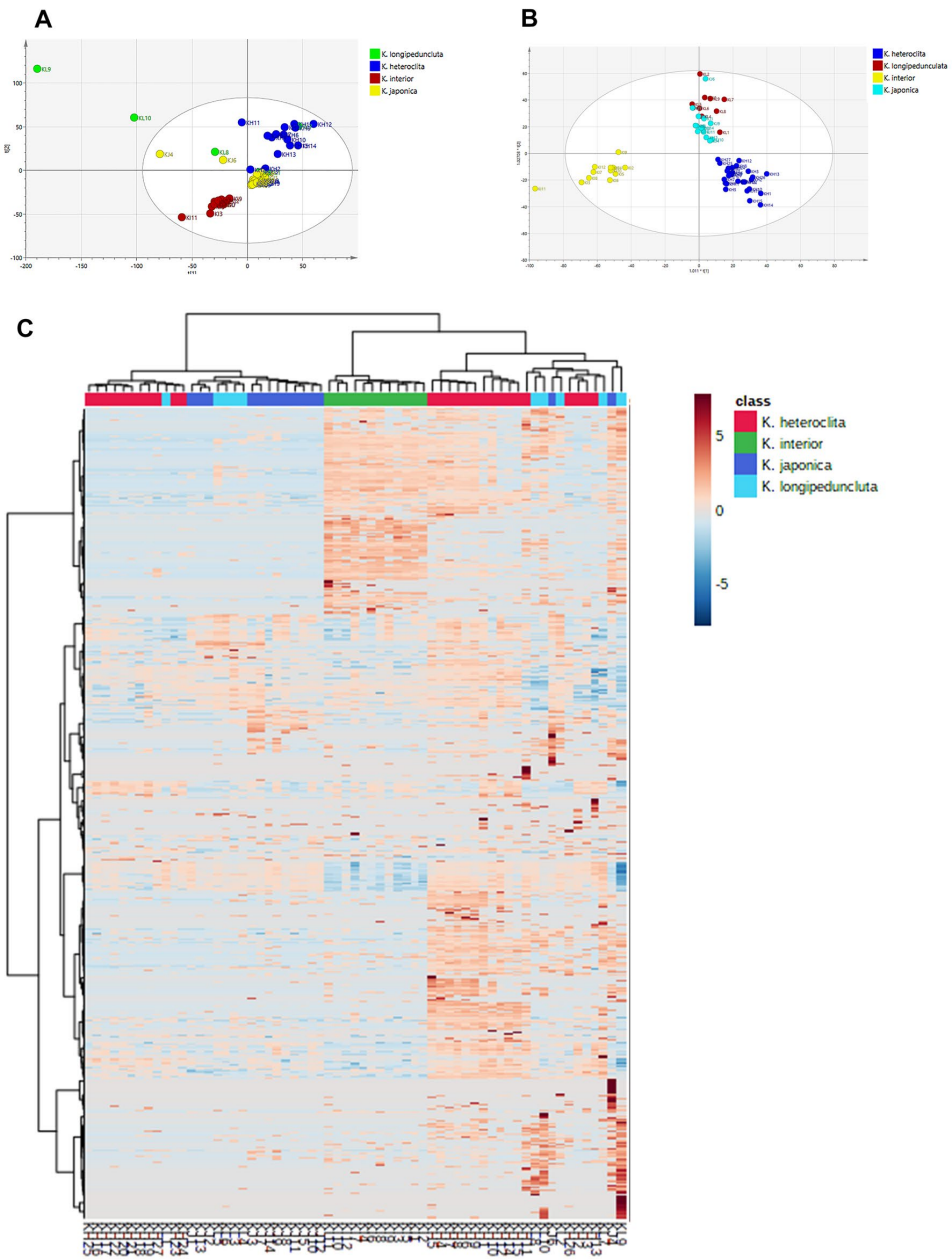
**A** PCA score plot derived from UPLC-Q/TOF–MS/MS datasets of *K. interior* and other three crude drugs. **B** OPLS-DA score plot derived from UPLC-Q/TOF–MS/MS datasets of *K. interior* and other three crude drugs. **C** Heatmap of the metabolite profiles of *K. interior* and other three crude drugs. The legend on the right and top indicates the grouping of metabolites and sample groups, respectively

The data quality control was analyzed with SIMCA (Additional file 1: Fig. S2–S3). To classify and differentiate the chemical constituents of four *Kadsura* crude drugs, PCA and OPLS‐DA were performed. PCA uses dimensionality reduction to transform multiple indicators into several composite metrics while maintaining the characteristics in the data that contribute the most to the variance. Data analysis was performed with Progenesis QI software for pre-treatment, including peak identification, peak alignment, normalization, and multivariate statistical analysis. The parameters used to assess the quality of the PCA model are R^2^ (cum) and Q^2^ (cum), with values close to 1.0 indicating good fitness and predictive power. This suggested that *K. interior* differed significantly from the other three crude drugs in chemical constituents (Fig. 6A).

**Fig. 7:**
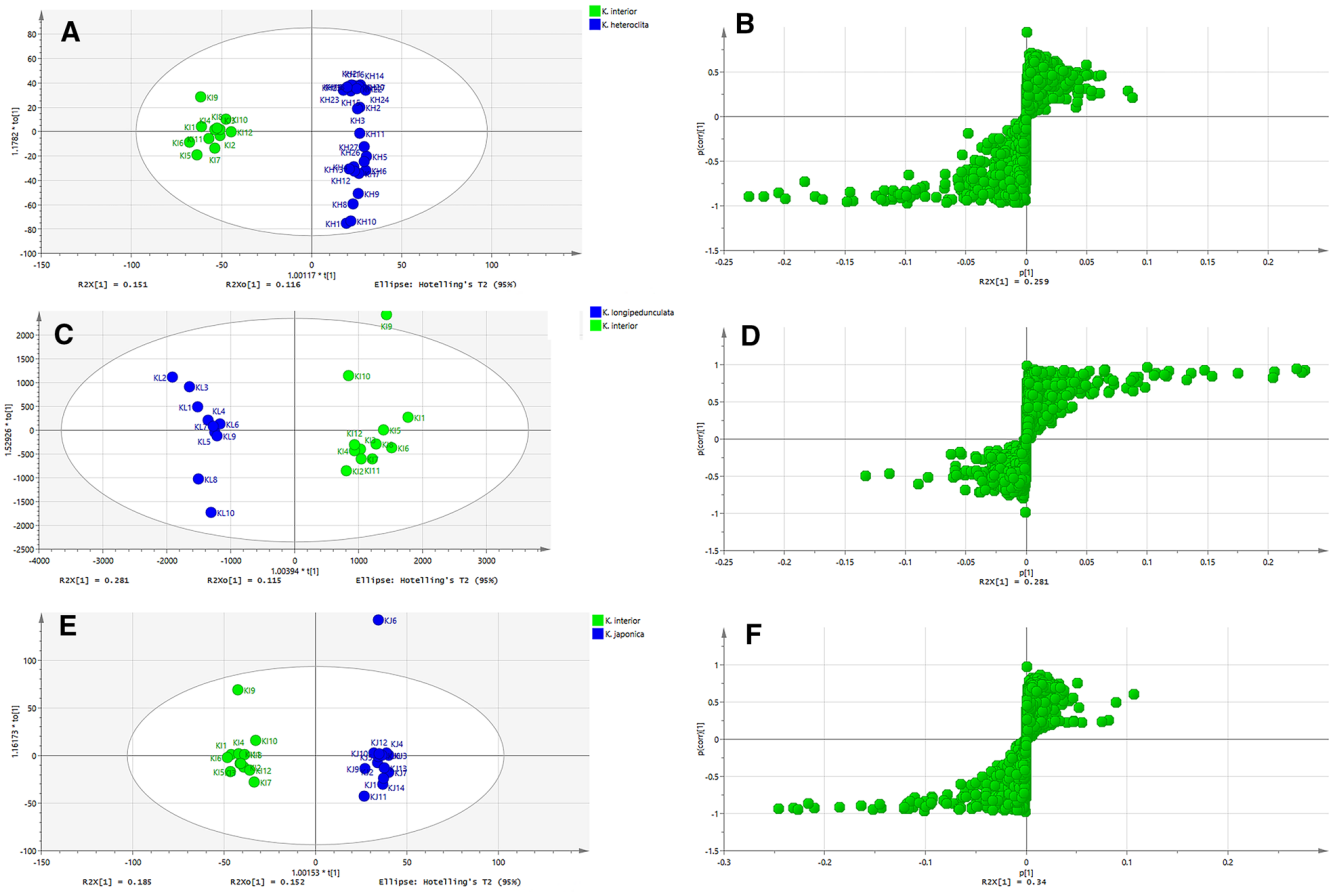
**A–B** OPLS-DA score plot and S-Plot of *K. interior*, *K. heteroclita*. **C–D **OPLS-DA score plot and S-Plot of *K. interior*, *K. longipedunculata*. **E–F** OPLS-DA score plot and S-Plot of *K. interior*, *K. japonica*

Based on the above results, a supervised OPLS-DA model was developed to find marker compounds of *K. interior*, which allows maximum differentiation of groups and facilitates the search for differential chemical compounds compared to PCA. In the score plot of the OPLS-DA model, *K. interior* could be separated with *K. heteroclita*, *K. longipedunculata*, and *K. japonica*. In addition, *K. longipedunculata* and *K. japonica* were mixed into one group, suggesting that these two crude drugs were hard to distinguish by chemical constituents (Fig. 6B). The heatmap (Fig. 6C) analysis of these four *Kadsura* crude drugs also showed that *K. interior* was clustered into one branch while the other three crude drugs were difficult to distinguish. To understand the characteristic marker compounds that have the greatest influence on the differences between the two groups, the main differential components were collected by S-plot. In S-plot, each point represents an exact mass-retention time (EMRT) pair, and the further away from the center point, the greater the influence of that point on the differences between groups, so EMRT pairs with high VIP values distributed at both ends of the S-plot were considered as potential characteristic marker compounds (Fig. 7). Our identification of ions at both ends of the S-plot summarized the differential compounds between *K. interior* and the other three species, separately. There were ten, six, and nine compounds that could be used to distinguish *K. interior* from *K. heteroclita*, *K. longipedunculata*, and *K. japonica* respectively. Six common compounds could be the chemical markers of *K. interior*: angeloylgomisin R, interiorin D, heteroclitin D, kadsurin, heteroclitin G, and heteroclitin E (Table 4). These marker compounds could be applied to identify *K. interior* from its closely related species.

**Table 4:** Differential compounds of *K. interior* distinguished from other three species

No	Compound name	vs *K. heteroclita*	vs *K. longipedunculata*	vs *K. japonica*
1	Heteroclitin G*	√	√	√
2	Angeloylgomisin M1	√		
3	Heteroclitin E*	√	√	√
4	Benzoyl Oxokadsuranol	√		
5	Acetoxyl Oxokadsurane			√
6	Interiotherin B	√		
7	Angeloylgomisin R*	√	√	√
8	Interiorin D*	√	√	√
9	Kadlongilignan E	√		
10	Heteroclitin D*	√	√	√
11	Kadsurin*	√	√	√
12	Schiarisanrin D			√
13	Schizandrin			√
Total number		10	6	9

Compounds marked with * could consistently distinguish *K. interior* from other three species

Compounds marked with √ showed they could distinguish *K. interior* from the species in corresponding column

#### Spectrum-effect relationship analysis

In this study, the spectrum-effect relationship based on chemical constituents of UPLC-Q/TOF–MS fingerprint and the blood tonic pharmacological indicators was analyzed with BCA and OPLSR to filter the compounds related to blood tonic activity. BCA was applied for the linear relationship between variables. The Pearson method was selected to calculate the correlation coefficient in this study. Compounds with Pearson’s correlation coefficient (r) of more than 0.500 were considered blood tonic activity-directly related within *K. interior* (Table 5). Six OPLSR models were established respectively with six blood tonic activity-related indicators (RBC, HGB, HCT, IL-3, GM-CSF, M-CSF) for the screening of blood tonic activity-directly related compounds (Y1: R_2_cum = 0.774633, Q_2_cum = 0.457938; Y2: R_2_cum = 0.971944, Q_2_cum = 0.855608; Y3: R_2_cum = 0.0657572, Q_2_cum = − 0.445958; Y4: R_2_cum = 0.0459692, Q_2_cum = − 0.482602; Y5: R_2_cum = 0.985895, Q_2_cum = 0.625163; Y6: R_2_cum = 0.36284, Q_2_cum = 0.282097), and the data were Pareto-scaled. The models established by Y1, Y2 and Y5 are available for further analysis (R_2_cum > 0.5, Q_2_cum > 0.2). In these models, compounds with VIP value over 1.0 and regression coefficient (b) over 0 were considered as blood tonic activity-directly related compounds in *K. interior* [28]. According to the above rules, three compounds (Heteroclitin G, Heteroclitin D, Interiorin C) were screened out, which were potential active markers within *K. interior* directly-related to the improvement of hematopoietic functions, and might be the key bioactive markers within *K. interior*. In addition, we performed quantitative analysis of two key bioactive components, which showed that KIS freeze-dried powder contained 15.90 and 3.74 μg/mg of heteroclitin D and heteroclitin G  (Additional file 1: Table S4), respectively, providing clues for future monomeric efficacy studies.

**Table 5:** Correlation coefficients of the chemical composition variable groups and the pharmacological variable groups with VIP values of the compounds

Compound	RBC#		HGB#		HCT		IL-3		GM-CSF#		M-CSF	
	BCA(r)	OPLSR (VIP)	BCA(r)	OPLSR (VIP)	BCA(r)	OPLSR (VIP)	BCA(r)	OPLSR (VIP)	BCA(r)	OPLSR (VIP)	BCA(r)	OPLSR (VIP)
**Heteroclitin G**	0.828	2.03	0.629	1.8594	0.252	2.0996	− 0.011	1.9421	0.7609	2.2051	0.6907	2.4739
Kadsutherin H	0.7842	0.1718	0.6386	0.1726	0.1364	0.1765	0.1298	0.165	0.6177	0.1765	0.6281	0.0188
**Interiorin C**	0.614	1.3025	0.858	1.4406	− 0.0287	1.2397	0.2238	1.3791	0.5701	1.3197	0.3061	0.7900
Kadsurin	0.4793	0.052	0.911	0.0578	− 0.1161	0.051	0.4774	0.0533	0.3995	0.0594	0.4301	0.0018
**Heteroclitin D**	0.6984	1.7646	0.82	1.834	0.0132	1.7267	0.1845	1.8059	0.5106	1.5206	0.4530	1.5014
Interiotherin C	0.6508	0.1504	0.6285	0.1502	0.2163	0.1507	0.0074	0.1497	0.5891	0.1489	0.5541	0.0129
Manwuwezic acid	0.1883	0.0903	0.6403	0.1319	− 0.0257	0.0784	0.1298	0.1051	0.2808	0.0995	0.0498	0.0063
Prinsepiol	0.4824	0.0685	− 0.0307	0.1698	− 0.0058	0.0966	− 0.1328	0.0313	0.2166	0.1232	0.6468	0.0313
Licarin A	0.633	0.0325	0.185	0.0408	0.365	0.0377	− 0.254	0.0265	0.7739	0.0448	0.6280	0.0018

Compounds marked with bold text are with VIP value greater than 1 and Pearson correlation coefficients (r value) greater than 0.5, which could be considered as significant contribution to the classification to be used as an evaluation index for pharmacology. The OPLSR models built to represent these pharmacological indicators (#) are available

## Discussion

*K. interior* is the original plant of Kadsurae Caulis, which has been utilized medicinally to tonify and invigorate the blood by traditional Chinese therapists for long periods. Previous phylogenetic systematics research revealed that three species (*K. heteroclita*, *K. longipedunculata*, and *K. japonica*) had a relatively close relationship with *K. interior*. Apart from the similarity in the genetic patterns, the distinction between *K. interior* and these three *Kadsura* species is further complicated for their similar morphological characteristics, which causes their misuse as Kadsurae Caulis frequently. The stems of these species are traditionally known for other therapeutic properties that differ from that of *K. interior*. The stems of *K. heteroclita* and *K. longipedunculata* possess the effects of expelling wind-evil and removing damp-evil, while the stems of *K. japonica* have antipyretic and pain-relieving properties [29, 30]. However, there was no report on the comparison of blood tonic efficacies and chemical constituents between the stems of *K. interior* and its closely related species.

In current study, the blood tonic effects of the stems of *K. interior* and its related species were compared with the BD mouse model. In clinical practice of TCM, BD is a disease characterized by pale face and lips, massive blood loss, a defective spleen, and poor hematogenesis [14]. In modern medicine, the symptoms of anemia are similar to those of BD, common clinical tests for anemia such as blood routine analysis are also used to aid in the diagnosis of BD [31]. Otherwise, hematopoietic growth factors comprise cytokines that influence blood cell growth and differentiation and are used to evaluate the hematopoietic function: IL-3 regulates the growth and production of major blood cell types [32], GM-CSF demonstrates proliferation activity on hematopoietic progenitor cells [33], M-CSF regulates the proliferation, differentiation, and survival of haemopoietic progenitor cells, especially in monocytes and macrophages [34]. These above indicators are generally used for the evaluation of the blood tonic efficacy of other herbal medicines [35, 36]. Hence, the body weights, the levels of blood routine indicators like HCT, HGB, and RBC, the thymus and spleen indexes, and the serum levels of hemopoietic growth factors, including IL-3, GM-CSF, and M-CSF, were adopted to evaluate the hematopoietic function [37, 38]. FEJ is a well-known and clinically effective proprietary Chinese medicine for the treatment of BD and therefore its clinical dose was used in the positive control of this study [39]. The observation and body weights of mice were only general indicators, the efficacies of blood tonic should be assessed primarily by reference to the data of blood routines. The results showed that *K. interior* could greatly increase the levels of HCT, HGB, RBC, the thymus index, and significantly decrease the spleen index, which is comparable to FEJ in terms of blood tonic efficacy. Moreover, at the low dose (200 mg/kg), KIS improved BD-relevant indicators like HGB and the thymus index more significantly than KLS and KJS, and was better than KHS. With dose conversion, 200 mg/kg was found to be close to the clinical dose of KIS [4], suggesting that KIS is the most effective at the clinical dose compared to its closely related species. Based on the findings in current study, the misuse of closely related species of *K. interior* as Kadsurae Caulis should be avoided. Additionally, *K. interior* was found to considerably regulate the serum levels of IL-3, GM-CSF, and M-CSF in BD mice. Among them, the trend in efficacy of KIS on blood routine indicators is consistent with that of M-CSF, while which is different from those of IL-3 and GM-CSF. The above results suggested that *K. interior* might contribute to hematopoietic function via M-CSF production and partly via IL-3/GM-CSF receptors [29]. The future study on the mechanism for blood tonic efficacy of KIS could prioritize the pathway including M-CSF and IL-3/GM-CSF.

Since the pharmacological properties of medicinal herbs are strongly associated with the chemical constituents, chemical analysis and identification of these *Kadsura* species are essential for exploring their therapeutic differences. In this study, an efficient extraction method and an optimized UPLC-Q/TOF–MS/MS analytical method for these *Kadsura* crude drugs were firstly established, and the comprehensive identification of chemical constituents in these crude drugs was performed using a plant metabolomics approach. The results showed that there were significant differences in the chemical constituents of *K. interior* and its closely related species. Furthermore, 20 compounds were identified with standard substances, 36 compounds were inferred with the in-house database, 70 compounds were identified with the public compounds database, and six common differential compounds of *K. interior* that could distinguish it from its closely related species, including angeloylgomisin R, interiorin D, heteroclitin D, kadsurin, heteroclitin G, and heteroclitin E, were selected with the S-plots.

In recent years, the spectrum-effect relationship has been successfully applied to evaluate the bioactive material basis, with common data processing methods such as principal component analysis (PCA), canonical correlation analysis (CCA), gray correlation analysis (GRA), bivariate correlation analysis (BCA), and orthogonal partial least-squares regression analysis (OPLSR) [18, 40]. Among them, the OPLSR method has high applicability when the datasets were small [41]. Therefore, in this study, the chemical constituents in UPLC-Q/TOF–MS fingerprint of the *K. interior* were combined with pharmacological data of blood tonic to perform BCA and OPLSR for spectrum-effect relationship analysis. The results showed that heteroclitin G, interiorin C, and heteroclitin D were the potential bioactive markers in *K. interior* related to the improvement of hematopoietic functions. Previous studies have reported that both heteroclitin D and heteroclitin G possess anti-oxidant activity and anti-lipid peroxidant activity [42, 43], whereas no research on the efficacy of interiorin C has been reported until now. Modern pharmacological studies have attributed the blood tonic efficacies of TCM to their anti-oxidant and immunomodulatory activities [44]. Anti-oxidants were reported to exert a protective effect on bone marrow nucleated cells (BMNCs). For example, hydrogen-rich water could increase the number of BMNCs and improve their self-renewal and proliferative capacity [45, 46]. The number of BMNCs is often considered as a direct indicator of the hematopoietic function of the bone marrow [47]. The bone marrow is a major hematopoietic organ and is an important source for the production of hematopoietic progenitor cells (HPCs). In this study, KIS was found to promote increasing the numbers of BMNCs in BD mice (Additional file 1: Fig. S4). Therefore, the anti-oxidant compounds such as heteroclitin D and heteroclitin G may be the key bioactive components. In addition, among these three potential bioactive markers, heteroclitin G and heteroclitin D are also the chemical markers that distinguish *K. interior* from its closely related species, which to some extent confirms the validity of the spectrum-effect relationship analysis. In the current Chinese pharmacopoeia (2020 edition, volume I), heteroclitin D is the only chemical compound to evaluate the quality of *K. interior* [4]. Nevertheless, this study indicated that heteroclitin G with medicinal properties should also be taken into consideration as another evaluation index, and its quantitative study should be carried out for further standard upgrading of Kadsurae Caulis. In addition, the potential bioactive markers mined from the spectrum-effect relationship analysis still require further pharmacological studies of blood tonic efficacy. In the following stage, we plan to carry out the pharmacological studies of heteroclin D, interiorin C, and heteroclitin G.

## Conclusion

This study showed that KIS exerted more advantageous blood tonic activity at the clinical dose, suggesting that the closely related species of *K. interior* should not be misused as Kadsurae Caulis. More attention should be paid to ensure that the original plant of Kadsurae Caulis in practical applications. 126 compounds in the stems of *K. interior* and its closely related species were comprehensively identified. Six differential compounds were pinpointed to distinguish *K. interior* from the other three closely related species, which could be regarded as marker compounds for *K. interior*. Afterwards, heteroclitin G, interiorin C, and heteroclitin D were uncovered as potential bioactive markers for the blood tonic activity of KIS with the spectrum-effect relationship analysis, the quantitative analysis of which provided a research basis for further pharmacological study. This study also provides research direction for the future study on the blood tonic pharmacological mechanism of Kadsurae Caulis.

## Supplementary Information

**Additional file 1**. Part I. The development of extraction and UPLC method. Part II. Data quality control. Part III. Effect of KIS on the bone marrow nucleated cell in BD mice. Part IV. All Compounds Identified from KIS, KHS, KLS, and KJS Based on Progenesis QI Software with public library. Part V. Quantitative of potential active ingredients

## Data Availability

The research data generated from this study are included in the article and additional files.

## Abbreviations

TCM: Traditional Chinese medicine
KIS: The stems of *K. interior*
KHS: The stems of *K. heteroclita*
KLS: The stems of *K. longipedunculata*
KJS: The stems of *K. japonica*
BD: Blood deficiency
UPLC-Q/TOF–MS/MS: Ultra-performance liquid chromatography-quadrupole-time of flight-mass spectrometry
ESI: Electrospray ionization
TIC: Total ion current
APH: 1-Acetyl-2-phenylhydrazine
CTX: Cyclophosphamide
s.c.: Subcutaneously
i.p.: Intraperitoneally
FEJ: Fufang E’jiao Jiang
CMC-Na: Carboxymethylcellulose sodium
EDTA: Ethylene diamine tetraacetic acid
HCT: Hematocrit
HGB: Hemoglobin
RBC: Red blood cells
ELISA: Enzyme-linked immunosorbent assay
IL-3: Interleukin 3
GM-CSF: Granulocyte–macrophage colony-stimulating factor
M-CSF: Macrophage-stimulating factor
ANOVA: Analysis of variances
SD: Standard deviation
EMRT: Exact mass-retention time
PCA: Principal component analysis
OPLSR: Orthogonal partial least-squares regression analysis
OPLS-DA: Orthogonal partial least squares discriminant analysis
BCA: Bivariate correlation analysis
HCA: Hierarchical cluster analysis
VIP: Variable importance in the projection
HPCs: Hematopoietic progenitor cells
BMNCs: Bone marrow nucleated cells
