# Supplementary material for: Comparison of blood tonic efficacy and chemical constituents of Kadsura interior A.C. Smith and its closely related species
Source: Chin Med. 2022 Jan 17;17:14. doi: 10.1186/s13020-021-00544-w (PMC8762946; doi:10.1186/s13020-021-00544-w)
Supplement: Supplementary file 1 — Additional file 1. Part I. The development of extraction and UPLC method. Part II. Data quality control. Part III. Effect of KIS on the bone marrow nucleated cell in BD mice. Part IV. All Compounds Identified from KIS, KHS, KLS, and KJS Based on Progenesis QI Software with public library. Part V. Quantitative of potential active ingredients [file 13020_2021_544_MOESM1_ESM.docx]

**Supplementary information**

**Comparison of blood tonic efficacy and chemical constituents of *Kadsura interior* A.C. Smith and its closely related species**

Jing Xu^1^, Jiushi Liu^1,2^, Bin Li^1,2^, Xueping Wei^1,2^, Yaodong Qi^1,2^, Bengang Zhang^1,2^, Haitao Liu^1,2*^, Peigen Xiao^1,2^

*^1^ Key Laboratory of Bioactive Substances and Resources Utilization of Chinese Herbal Medicine**, Ministry of Education, Institute of Medicinal Plant Development, Chinese Academy of Medical Sciences, Peking Union Medical College, Beijing 100193, China*

*^2^ Engineering Research Center of Traditional Chinese Medicine Resource, Ministry of Education, Institute of Medicinal Plant Development, Chinese Academy of Medical Sciences, Peking Union Medical College, Beijing, China*

***Correspondence Author:**

Professor Haitao Liu

E-mail: [htliu0718@126.com](mailto:htliu0718@126.com)

Fax number: 010-57833196

**Contents**

**Part I. The development of extraction and UPLC method**

**Part II. Data quality control**

**Part III. Effect of KIS on the bone marrow nucleated cells in BD mice**

**Part IV. All Compounds Identified from KIS, KHS, KLS, and KJS Based on Progenesis QI Software with public library**

**Part V. Quantitative of potential active ingredients**

# Part I. The development of extraction and UPLC method

### 1. Optimization of extraction procedures

An orthogonal experiment design method (L_9_ 3^3^) was employed to analyze the effects of solvent concentrations and ultrasound time on the extraction efficiency of KIS (Table S1). The air-dried KIS was crushed and passed through a 60-mesh sieve. 0.5000 g powdered KIS samples were extracted according to nine groups of different conditions and the loss of weight was compensated with extraction solvent. The supernatant was filtered through a 0.22 μm membrane filter.

**Table S1** Experimental layout based on an L_9_ orthogonal array

| **No** | **A（Material-liquid ratios）** | **B（Solvent concentrations）** | **C （Ultrasound time）** |
| --- | --- | --- | --- |
| 1 | 1:15 | 70% methanol | 15 min |
| 2 | 1:15 | 85% methanol | 30 min |
| 3 | 1:15 | 100% methanol | 60 min |
| 4 | 1:30 | 70% methanol | 15 min |
| 5 | 1:30 | 85% methanol | 30 min |
| 6 | 1:30 | 100% methanol | 60 min |
| 7 | 1:45 | 70% methanol | 15 min |
| 8 | 1:45 | 85% methanol | 30 min |
| 9 | 1:45 | 100% methanol | 60 min |

The measured peak area of one chemical component was used as the inspection index. The effects of each factor in the extraction of KIS were as follow: the extraction solvent, the ratio of material to liquid, the number of ultrasounds, the results of the intuitive analysis were shown in Table S2. Based on comprehensive considerations, the best extraction process of KIS was A_1_B_2_C_2_, that is, the samples (0.5000 g, 60 mesh) were accurately weighed and extracted with 7.5 mL 85% methanol by ultra-sonication (35 kHz) for 30 min. The supernatant was filtered through a 0.22 μm membrane prior to injection into the UPLC system.

**Table S2** The orthogonal test arrangement and intuitive analysis（n=3）

| **No.** | **A (solid-liquid ratio)** | **B (extraction solvent)** | **C (ultrasonic time)** | **Maximum peak area** |
| --- | --- | --- | --- | --- |
| 1 | 1 | 1 | 1 | 4346406 |
| 2 | 1 | 2 | 2 | 4535722 |
| 3 | 1 | 3 | 3 | 4514880 |
| 4 | 2 | 1 | 2 | 2191227 |
| 5 | 2 | 2 | 3 | 2095704 |
| 6 | 2 | 3 | 1 | 2078347 |
| 7 | 3 | 1 | 3 | 1343562 |
| 8 | 3 | 2 | 1 | 1338172 |
| 9 | 3 | 3 | 2 | 1354364 |
| K1 | 4465669.333 | 2627065.000 | 2587641.667 |  |
| K2 | 2121759.333 | 2656532.667 | 2693771.000 |  |
| K3 | 1345366.000 | 2649197.000 | 2651382.000 |  |
| R | 3120303.333 | 29467.667 | 106129.333 |  |

### 2. Optimization of the chromatographic conditions of UPLC

Since the constituents of four *Kadsura* herbs are mainly lignans and triterpenoids, three C18 reversed chromatography columns, BEH C18, CSH C18 and CORTECS C18, which are suitable for the separation of non-polar or weakly polar compounds, were used in this study.

To obtain abundant chromatographic information of *K. interior*, we observed the chromatographic separation effects with different chromatographic columns (ACUITY BEH C18, ACUITY CSH C18, CORTECS C18), different column temperatures (25 ℃, 30 ℃, 40 ℃), different flow rates (0.2 mL/min, 0.3 mL/min, 0.4 mL/min), different mobile phases (methanol, acetonitrile) and different elution gradients with Agilent 1290 UPLC System. The relative results could be seen at ‘UPLC-Q/TOF-MS/MS conditions’ of the main body.

The chromatographic peaks showed that more chromatographic information could be obtained using CORTECS C18 (Fig. S1A). Two typical organic phases were selected as the mobile phase: methanol and acetonitrile. Among them, when acetonitrile was used as the mobile phase, the compound could be eluted more uniformly during 16-20 min (Fig. S1B). In addition, the effects of three column temperatures and three flow rates on chromatographic separation were investigated in this study. The results found that the peaks were best separated at 25 ℃ and at a flow rate of 0.3 mL/min for 12-14 min and 26-28 min (Fig. S1C-D). The detection wavelength of the PDA detector was set to 215 nm (Fig. S1E).

The final optimized chromatographic analysis conditions were as follows:

Column: Waters UPLC CORTECS C18 (2.1×100 mm, 1.6 μm); column temperature: 25 ℃; mobile phase: water (A), acetonitrile (B); flow rate: 0.3 mL/min; injection volume: 1μL; detection wavelength: 215 nm; binary system gradient elution program: 0-4 min, 33% B→36% B; 4-5 min, 36% B→45% B; 5-9 min, 45% B; 9 -12 min, 45% B→50% B; 12-16 min, 50% B→56% B; 16-22 min, 56% B→70% B; 22-30 min, 70% B→95% B. This condition is also applicable to Waters ACQUITY UPLC™ system.

**
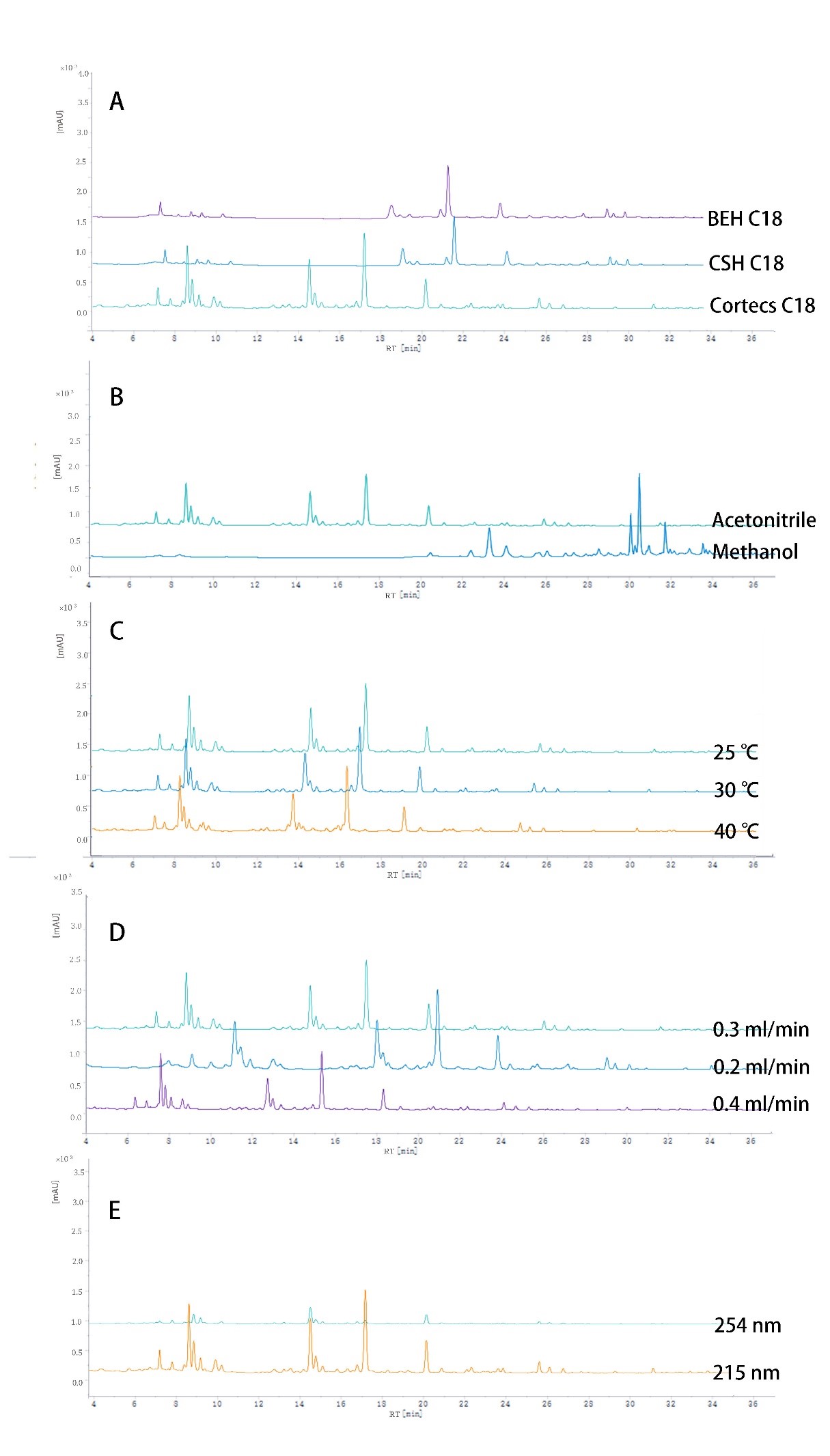
**

**Fig. S1.** UPLC chromatographic conditions optimization graphs

A: chromatographic columns; B: mobile phases; C: column temperatures; D: flow rates; E: wavelengths

# Part II. Data quality control

To obtain real and reliable data, data quality control was performed in this study from the following two aspects: first, in the positive ion mode of UPLC-Q/TOF-MS/MS analysis, the detection was carried out by interspersed sampling between different groups and random sampling within groups. second, the stability of the detection system was examined by plotting the PCA score graph of quality control (QC) samples and the retention time variation graphs of all the actual samples (Fig. S2). Taking the PCA score graph constructed for QC samples and the retention time variation plots of the actual sample as an example, the peak surface of all QC samples deviation is between -2SD to 2SD. The results showed that the separation performance and stability of the chromatographic system are good, and the analytical method was stable and reliable, which guarantees the differences between groups obtained by data processing come from the differences in metabolites of *K. interior* and its closely relative species, rather than human errors or instrumental system errors (Fig. S3).

**
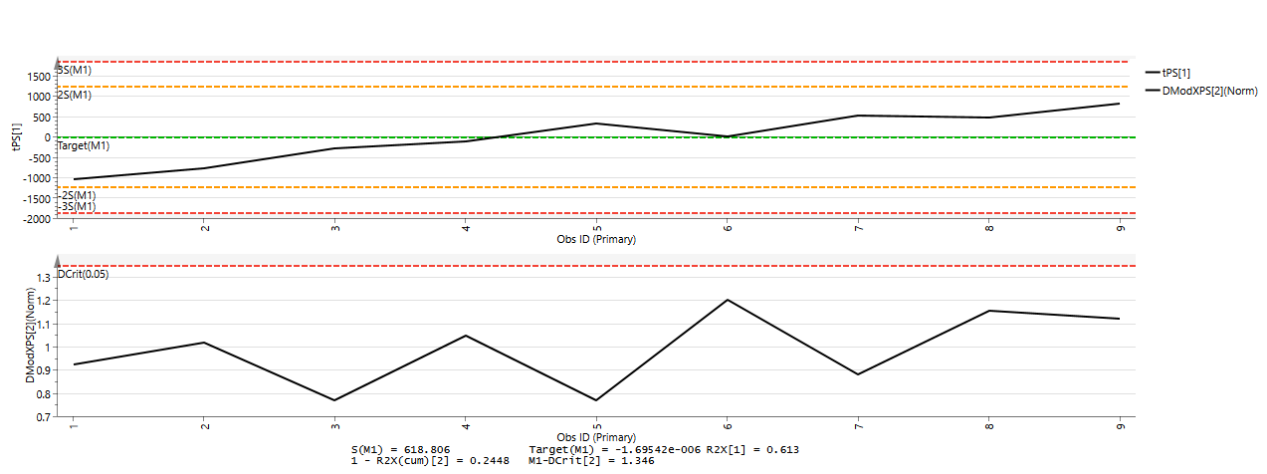
**

**Fig. S2** Principal component analysis (PCA) score plot of quality control (QC) samples**
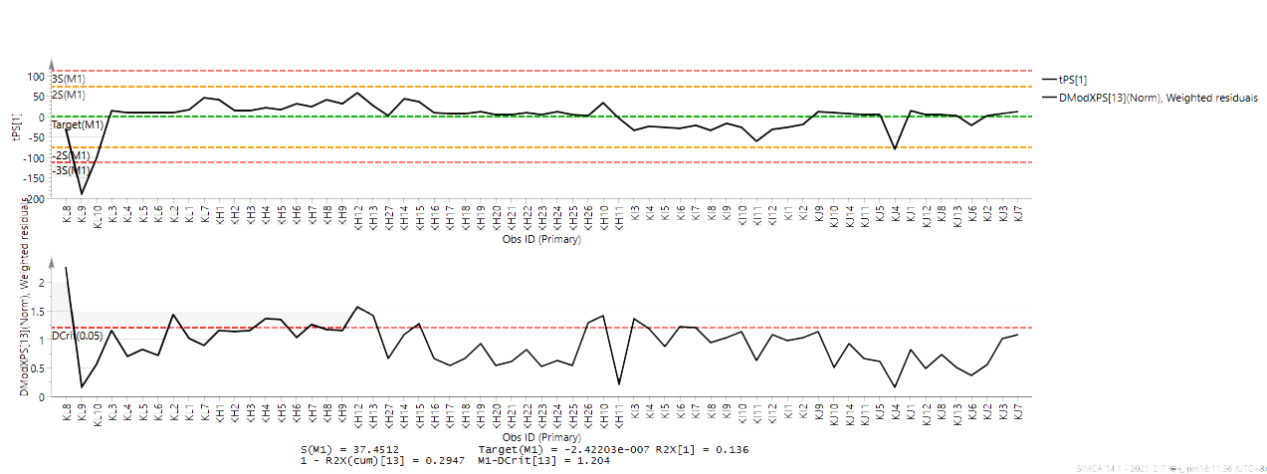
**

**Fig. S3** Principal component analysis (PCA) score plot of real samples

# Part III. Effect of KIS on the bone marrow nucleated cells in BD mice

Bone marrow nucleated cells (BMNCs) of mice in model group were reduced significantly compared to that in control group (*P* < 0.01). While BMNCs of mice in KIS extract groups were all increased evidently compared to mice in model group (*P* < 0.001) (Fig. S4).

**
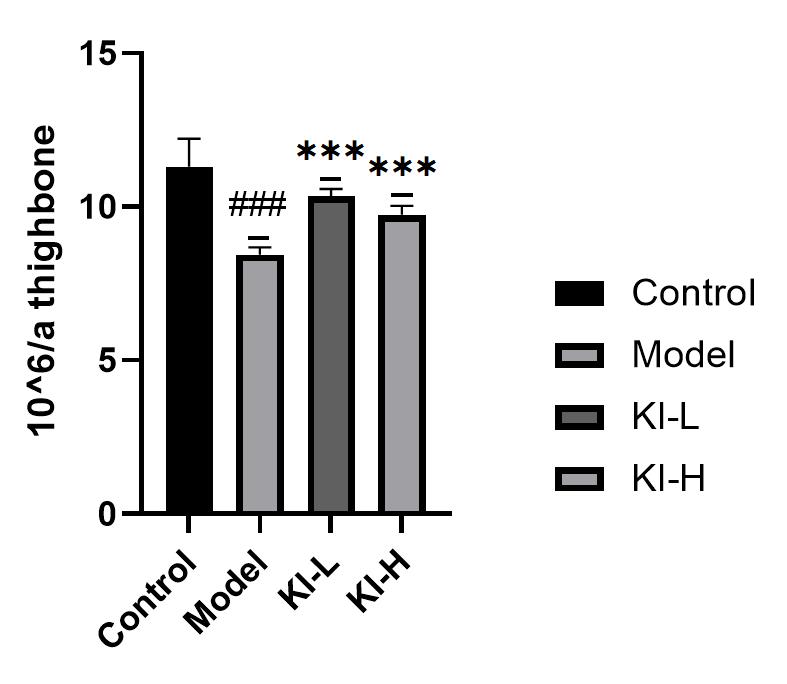
**

**Fig. S4** Effect of KIS on BMNCs of BD-mice (mean±SD, n=10). ^###^*P <* 0.001 vs control group, ****P* < 0.001 vs model group

# Part IV. All Compounds Identified from KIS, KHS, KLS, and KJS Based on Progenesis QI Software with public librar

**Table S3 All Compounds Identified from KIS, KHS, KLS, and KJS Based on Progenesis QI Software with public library**

| **No.** | **t_R_-*m/z*** | **Adducts** | **Formula** | **Score** | **FS** | **ME** | **IS** | **Compound ID** | **MFC** | **Highest Mean** | **Lowest Mean** |
| --- | --- | --- | --- | --- | --- | --- | --- | --- | --- | --- | --- |
| **Alkaloids** | |  |  |  |  |  |  |  |  |  |  |
| 1 | 1.41_128.1438m/z | M+H | C_8_H_17_N | 38 | 0 | 2.95 | 93.27 | DL-Coniine | 1.97 | KHS | KIS |
| 2 | 10.46_632.3061m/z | M+H | C_33_H_45_NO_11_ | 37.1 | 0.26 | -0.7 | 86.3 | Mesaconitine | 12.94 | KLS | KHS |
| 3 | 12.57_603.1793m/z | 2M+Na | C_11_H_18_N_2_O_5_S | 37.6 | 5.08 | 4.8 | 88.65 | Glutamyl-S-allylcysteine | Infinity | KLS | KIS |
| 4 | 16.69_369.1929n | M+H, M+Na | C_22_H_27_NO_4_ | 38.6 | 0.13 | -2.99 | 96.36 | (+)-Corydaline | 36.05 | KJS | KIS |
| 5 | 16.69_373.1642m/z | 2M+H | C_11_H_10_N_2_O | 35 | 0.39 | -4.48 | 79.65 | L-Oxonoreleagnine | 12.94 | KLS | KIS |
| 6 | 19.58_687.1742m/z | 2M+Na | C_20_H_14_NO_4_ | 38.4 | 0.07 | 0.58 | 92.85 | Sanguinarine | 1.00 | --- | --- |
| 7 | 19.62_668.3064m/z | M+Na | C_34_H_47_NO_11_ | 35.9 | 0.95 | 3.45 | 82.76 | Aconitine | 4.72 | KJS | KHS |
| 8 | 20.01_490.2798m/z | M+Na | C_25_H_41_NO_7_ | 38.5 | 0.03 | 4.84 | 98.14 | Lycoctonine | Infinity | KHS | KIS |
| 9 | 21.52_331.2386m/z | 2M+H | C_10_H_15_NO | 37.2 | 0 | 1.9 | 88.29 | Hordenine | Infinity | KHS | KIS |
| 10 | 22.97_581.2386m/z | 2M+Na | C_18_H_17_NO_2_ | 35.3 | 0 | -4.42 | 81.55 | Remerine | Infinity | KHS | KLS |
| 11 | 3.22_616.3126m/z | M+H | C_33_H_45_NO_10_ | 36.3 | 0.78 | 1.52 | 82.67 | Hypaconitine | Infinity | KJS | KLS |
| 12a | 30.67_161.1073m/z | M+H | C_10_H_12_N_2_ | 38.5 | 0 | -0.16 | 92.92 | Anatabine (not validated) | 1.21 | KJS | KIS |
| 12b | 30.67_161.1073m/z | M+H | C_10_H_12_N_2_ | 38.5 | 0 | -0.16 | 92.92 | Tryptamine (not validated) | 1.21 | KJS | KIS |
| 13 | 4.27_387.1459m/z | M+Na | C_22_H_22_NO_4_ | 37.9 | 0 | 4.84 | 95.14 | Coralyne | 20.72 | KLS | KJS |
| 14 | 5.89_369.2042m/z | M+Na | C_22_H_26_N_4_ | 37.8 | 0.56 | -2.16 | 90.95 | Calycanthine | 27.25 | KHS | KIS |
| 15a | 6.78_513.1889m/z | M+H | C_26_H_28_N_2_O_9_ | 37.8 | 0.19 | 4.12 | 93.54 | Pumiloside | 86.23 | KLS | KHS |
| 15b | 6.78_513.1889m/z | M+H | C_26_H_28_N_2_O_9_ | 37.8 | 0.2 | 4.12 | 93.54 | Carboline metabolite (C_26_H_28_N_2_O_9_) | 86.23 | KLS | KHS |
| 15c | 6.78_513.1889m/z | M+H | C_26_H_28_N_2_O_9_ | 37.8 | 0.29 | 4.12 | 93.54 | Lyalosidic acid | 86.23 | KLS | KHS |
| 16 | 8.29_373.1641m/z | 2M+H | C_11_H_10_N_2_O | 35 | 0 | -4.87 | 80.68 | Deoxyvasicinone | 353.97 | KLS | KIS |
| 17 | 9.44_399.1795m/z | 2M+Na | C_11_H_12_N_2_O | 38.3 | 0 | 0.97 | 92.49 | Vasicine | 36.94 | KJS | KIS |
| **Coumarin** | |  |  |  |  |  |  |  |  |  |  |
| 18 | 6.81_267.0996m/z | M+Na | C_15_H_16_O_3_ | 29.9 | 0.19 | 1.94 | 51.68 | Osthole | 45.09 | KLS | KJS |
| **Fatty acid** | |  |  |  |  |  |  |  |  |  |  |
| 19 | 20.96_317.2093m/z | M+Na | C_18_H_30_O_3_ | 38.4 | 0 | 2.05 | 94.29 | FA 18:3+1O | Infinity | KHS | KLS |
| 20 | 13.83_293.2121m/z | M+H | C_18_H_28_O_3_ | 38.3 | 0 | 3.37 | 95.46 | FA 18:4+1O | Infinity | KLS | KIS |
| **Flavonoids** | |  |  |  |  |  |  |  |  |  |  |
| 21 | 0.65_657.1661m/z | M+H | C_28_H_32_O_18_ | 33.4 | 0 | -0.03 | 67.11 | Flavonol base + 4O, 1MeO, O-Hex-Hex | 4.14 | KLS | KIS |
| 22 | 0.75_611.1628m/z | M+H | C_27_H_30_O_16_ | 36.2 | 0.19 | 3.57 | 84.85 | Flavone base + 4O, C-Hex-Hex | 4.25 | KLS | KIS |
| 23 | 0.78_595.1468m/z | M+H | C_30_H_26_O_13_ | 35.5 | 0.43 | 3.65 | 81.25 | Flavonol base + 3O, O-Hex, O-coumaroyl | 4.70 | KLS | KIS |
| 24 | 12.21_315.0875m/z | M+H | C_17_H_14_O_6_ | 35.4 | 0 | 3.69 | 81.53 | Flavone + 2O + 2MeO | 25.12 | KIS | KHS |
| 25 | 12.60_299.0919m/z | M+H | C_17_H_14_O_5_ | 39.3 | 3.55 | 1.58 | 95.08 | Flavonol base + 2MeO | 33.78 | KIS | KJS |
| 26a | 12.60_325.1420m/z | M+H | C_20_H_20_O_4_ | 39.5 | 6.97 | -4.46 | 95.5 | Chalcone base + 3O, 1Prenyl | 97.98 | KIS | KJS |
| 26b | 12.60_325.1420m/z | M+H | C_20_H_20_O_4_ | 38.4 | 1.57 | -4.46 | 95.5 | Flavanone base + 2O, 1Prenyl | 97.98 | KIS | KJS |
| 27 | 16.72_677.3136m/z | 2M+H | C_21_H_22_O_4_ | 31.4 | 0.07 | 4.06 | 61.56 | Chalcone base + 2O, 1MeO, 1Prenyl or Licochalcone A (not validated) | 12.73 | KIS | KHS |
| 28 | 16.84_645.1746m/z | M+Na | C_36_H_30_O_10_ | 36.7 | 1.24 | 2.45 | 85.42 | Biflavonoid-flavone base + 3MeO and flavone base + 3MeO | Infinity | KHS | KLS |
| 29a | 20.37_355.1184m/z | M+H | C_20_H_18_O_6_ | 37.8 | 0 | 2.23 | 91.59 | Flavone base + 4O, 1Prenyl | 61.05 | KIS | KLS |
| 29b | 20.37_355.1184m/z | M+H | C_20_H_18_O_6_ | 37.8 | 0 | 2.23 | 91.59 | Isoflavone base + 4C, 1Prenyl | 61.05 | KIS | KLS |
| 30 | 24.98_537.1523m/z | 2M+H | C_16_H_12_O_4_ | 35.7 | 0 | -3.98 | 83.27 | Isoflavone base + 1O, 1MeO | Infinity | KLS | KIS |
| 31 | 25.68_285.0770m/z | M+H | C_16_H_12_O_5_ | 36 | 1.99 | 4.41 | 83.07 | Wogonin | Infinity | KHS | KLS |
| 32 | 32.15_521.1282m/z | M+H | C_24_H_24_O_13_ | 39.3 | 0.46 | -1.46 | 97.62 | Anthocyanidin base+3O, O-MalonylHex | 2.16 | KLS | KIS |
| 33 | 5.55_353.1391m/z | M+H | C_21_H_20_O_5_ | 40.1 | 8.9 | 2.25 | 94.33 | Isoflavone base + 2O, 1MeO, 1Prenyl | 13.76 | KIS | KJS |
| 34 | 5.55_355.1541m/z | M+H | C_21_H_22_O_5_ | 39.6 | 6.33 | 0.18 | 91.71 | Chalcone base + 3O, 1MeO, 1Prenyl | 495.35 | KIS | KJS |
| 35 | 6.47_368.1278n | M+H, 2M+Na | C_21_H_20_O_6_ | 38.8 | 6.43 | 4.82 | 93.27 | Flavone base + 3O, 1MeO, 1Prenyl | 21.77 | KIS | KJS |
| 36 | 7.31_323.1281m/z | M+H | C_20_H_18_O_4_ | 41.3 | 15.4 | 1.09 | 92.4 | Flavone base + 2O, 1Prenyl | 36.14 | KIS | KJS |
| 37 | 7.34_559.1339m/z | 2M+Na | C_16_H_12_O_4_ | 38.1 | 0.06 | -4.48 | 95.71 | Formononetin | 1775.12 | KIS | KJS |
| 38a | 9.01_357.1342m/z | M+H | C_20_H_20_O_6_ | 38.4 | 0 | 2.73 | 95.33 | Flavanone + 4O, 1Prenyl | 3.64 | KLS | KHS |
| 38b | 9.01_357.1342m/z | M+H | C_20_H_20_O_6_ | 38.4 | 0 | 2.73 | 95.33 | Isoflavanone base + 4O, 1Prenyl | 3.64 | KLS | KHS |
| 39 | 9.74_357.1343m/z | M+H | C_20_H_20_O_6_ | 39.4 | 6.19 | 2.96 | 94.35 | Flavanone base + 4O, 1Prenyl | 77.45 | KLS | KHS |
| **Glycoside** | |  |  |  |  |  |  |  |  |  |  |
| 40a | 0.75_611.1628m/z | M+H | C_27_H_30_O_16_ | 40.7 | 22.8 | 3.57 | 84.85 | Kaempferol 3-O-sophoroside | 4.25 | KLS | KIS |
| 40b | 0.75_611.1628m/z | M+H | C_27_H_30_O_16_ | 39.3 | 15.6 | 3.57 | 84.85 | Luteolin 6-C-glucoside 8-C-arabinoside | 4.25 | KLS | KIS |
| 41 | 0.78_595.1468m/z | M+H | C_30_H_26_O_13_ | 36 | 2.99 | 3.65 | 81.25 | Kaempferol-3-O-glucoside-6''-p-coumaroyl | 4.70 | KLS | KIS |
| 42a | 5.69_1189.3938m/z | 2M+H | C_28_H_34_O_14_ | 35.4 | 0.94 | -2.67 | 79.2 | Isosakuranetin-7-O-neohesperidoside | Infinity | KLS | KIS |
| 42b | 5.69_1189.3938m/z | 2M+H | C_28_H_34_O_14_ | 35.2 | 0.2 | -2.67 | 79.2 | Isosakuranetin-7-O-rutinoside | Infinity | KLS | KIS |
| 43 | 9.14_365.1038m/z | M+Na | C_12_H_22_O_11_ | 35 | 0 | -4.85 | 80.37 | Disaccharide(Hex-Hex) | 299.41 | KLS | KJS |
| 44 | 12.40_633.1766m/z | M+Na | C_28_H_34_O_15_ | 34.9 | 0.71 | -3.93 | 78.21 | Hesperetin-7-O-neohesperidoside | 916.64 | KJS | KHS |
| 45 | 13.79_635.1919m/z | M+Na | C_28_H_36_O_15_ | 37.7 | 3.98 | -4.54 | 90.02 | Dihydrohesperetin-7-O-neohesperidoside | Infinity | KJS | KIS |
| 46 | 15.04_497.2913m/z | 2M+H | C_15_H_20_O_3_ | 37.5 | 0.21 | 3.04 | 90.69 | Atractylenolide III | 3.66 | KJS | KHS |
| **Lignans** | |  |  |  |  |  |  |  |  |  |  |
| 47 | 5.52_415.1401m/z | M+H | C_22_H_22_O_8_ | 40.3 | 13.3 | 3.31 | 92.07 | (-)-Podophyllotoxin | 122.09 | KLS | KHS |
| 48 | 6.18_432.2152n | M+H, M+Na, 2M+Na | C_24_H_32_O_7_ | 49.3 | 54.1 | 0.95 | 93.51 | Schizandrin | 22.58 | KJS | KHS |
| 49 | 6.81_325.1423m/z | M+Na | C_18_H_22_O_4_ | 33.6 | 0.04 | 4.18 | 73.01 | Enterodiol | 221.62 | KLS | KJS |
| 50 | 6.81_359.1478m/z | M+H | C_20_H_22_O_6_ | 39.5 | 8.43 | -3.03 | 92.79 | Matairesinol | 122.52 | KLS | KHS |
| 51 | 10.46_373.1642m/z | M+H | C_21_H_24_O_6_ | 39.2 | 8.63 | -0.85 | 88.2 | Arctigenin | 167.45 | KLS | KIS |
| 52 | 12.24_455.1483m/z | M+H | C_28_H_22_O_6_ | 37.9 | 0.14 | -1.35 | 91.03 | Epsilon-Viniferin | 378.97 | KIS | KHS |
| **Lipids** | |  |  |  |  |  |  |  |  |  |  |
| 53 | 14.58_496.3418m/z | M+H | C_24_H_50_NO_7_P | 37.3 | 0.08 | 4.1 | 91.08 | LPC 16:0 | 2.86 | KLS | KIS |
| 54 | 14.54_520.3383m/z | M+H | C_26_H_50_NO_7_P | 35.5 | 3.22 | -2.75 | 77.66 | LPC 18:2 | 71838.46 | KLS | KIS |
| **Phenolic acid and derivatives** | | | |  |  |  |  |  |  |  |  |
| 55a | 9.01_357.1342m/z | 2M+H | C_10_H_10_O_3_ | 37.1 | 0 | 2.73 | 88.65 | 3-Methoxycinnamic acid | 3.64 | KLS | KHS |
| 55b | 9.01_357.1342m/z | 2M+H | C_10_H_10_O_3_ | 37.1 | 0 | 2.73 | 88.65 | Coniferyl aldehyde | 3.64 | KLS | KHS |
| 55c | 9.01_357.1342m/z | 2M+H | C_10_H_10_O_3_ | 37.1 | 0 | 2.73 | 88.65 | Methoxycinnamic acid | 3.64 | KLS | KHS |
| 56 | 14.81_541.2560m/z | 2M+H | C_17_H_18_O_3_ | 38.1 | 0.02 | -4.56 | 95.86 | E-Resveratrol trimethyl ether | 429.47 | KIS | KJS |
| 57 | 20.37_355.1184m/z | M+H | C_20_H_18_O_6_ | 37.8 | 0 | 2.23 | 91.59 | Triacetyl resveratrol | 61.05 | KIS | KLS |
| **Saponin** | |  |  |  |  |  |  |  |  |  |  |
| 58 | 12.53_965.5070m/z | M+Na | C_48_H_78_O_18_ | 35.4 | 0 | -1.07 | 78.47 | Soyasaponin Bb | 143.92 | KIS | KLS |
| **Terpenoids** | | | |  |  |  |  |  |  |  |  |
| 59 | 5.93_441.1405m/z | M+H | C_20_H_24_O_11_ | 38.2 | 0.02 | 3 | 94.61 | Ginkgolide C | 36.27 | KIS | KLS |
| **Others** | |  |  |  |  |  |  |  |  |  |  |
| 60 | 7.70_393.0960m/z | M+H | C_22_H_16_O_7_ | 37.4 | 0 | -2.19 | 89.49 | Jusmicranthin ethyl ether | Infinity | KHS | KIS |
| 61 | 22.22_495.1780m/z | M+H | C_26_H_26_N_2_O_8_ | 36.5 | 0 | 3.56 | 86.72 | Carboline metabolite (C26H26N2O8) | Infinity | KHS | KLS |
| 62 | 9.38_325.1427m/z | 2M+H | C_10_H_10_O_2_ | 36.1 | 0 | -2.32 | 83.36 | Methyl cinnamate | 67.74 | KLS | KIS |
| **Unknown structures** | | |  |  |  |  |  |  |  |  |  |
| 63a | 27.48_273.2336m/z | 2M+H | C_9_H_14_N | 38.5 | 0 | 3.82 | 96.81 | C_9_H_14_N | 2.37 | KHS | KLS |
| 63b | 27.48_273.2336m/z | 2M+H | C_9_H_14_N | 38.5 | 0 | 3.82 | 96.81 | C_9_H_14_N | 2.37 | KHS | KLS |
| 64 | 4.67_353.1034m/z | 2M+H | C_10_H_8_O_3_ | 38 | 0 | 4.2 | 95.1 | C_10_H_8_O_3_ | Infinity | KIS | KJS |
| 65 | 3.15_209.1531m/z | M+H | C_13_H_20_O_2_ | 36.7 | 0 | -2.56 | 86.41 | C_13_H_20_O_2_ | 56.54 | KHS | KLS |
| 66 | 3.25_205.0618m/z | M+Na | C_13_H_10_O | 36.6 | 0 | -3.24 | 86.69 | C_13_H_10_O | 12.61 | KJS | KLS |
| 67 | 14.87_239.0713m/z | M+H | C_15_H_10_O_3_ | 34.1 | 7.59 | 4.23 | 67.71 | C_15_H_10_O_3_ | 672.76 | KIS | KJS |
| 68 | 0.65_685.1984m/z | M+H | C_30_H_36_O_18_ | 33.6 | 0.87 | 1.47 | 69.05 | C_30_H_36_O_18_ | 2.72 | KHS | KIS |
| 69 | 4.21_181.1222m/z | M+H | C_11_H_16_O_2_ | 38.4 | 0 | -0.72 | 92.9 | C_11_H_16_O_2_ | 2.91 | KHS | KIS |
| 70 | 12.21_315.0875m/z | M+H | C_17_H_14_O_6_ | 35.4 | 0 | 3.69 | 81.53 | C_17_H_14_O_6_ | 25.12 | KIS | KHS |

positive mode; FS. (fragmental score); ME (mass error, ppm); IS (isotope similarity); a-c (designates isomers)

# Part V. Quantitative of potential active ingredients

To explore the blood tonic efficacies of potential bioactive compounds mentioned in the main body, the quantitative study of two potential bioactive compounds was conducted. Except for the interiorin C was not available in the market, standard compounds of heteroclitin D and heteroclitin G for the quantitative study were bought from RuiFenSi Biotechnology Co., Ltd. (Sichuan, China).

The method of quantitative analysis was evaluated in terms of linearity, limit of detection (LOD), and limit of quantification (LOQ). Each compound was analyzed by plotting the peak area (y) against its concentration (x, mg/mL) in the mixed standard solution, which was expressed as table S4.

**Table S4** Calibration curves, *r*, linear range, LOD and LOQ for two standard compounds

| No. | Compounds | Regression equation | Linear range (μg/mL) | *r* | LOD (μg/mL) | LOQ (μg/mL) |
| --- | --- | --- | --- | --- | --- | --- |
| 1 | Heteroclitin D | y = 26625x + 332.01 | 40-800 | 0.9991 | 13.11 | 43.64 |
| 2 | Heteroclitin G | y = 31886x + 216.53 | 20-400 | 0.9994 | 16.73 | 48.72 |

Using the regression equation, we found that the contents of heteroclitin D and heteroclitin G in the freeze-dried powder of KIS were 15.90 and 3.74 μg/mg, respectively. For future pharmacological experiment of heteroclitin D and heteroclitin G based on BD mouse model, it suggests the low and high doses of heteroclitin D could be set at around 3.18 and 6.36 mg/kg, and those of heteroclitin G could be set at around 0.75 and 1.50 mg/kg.
